# Ancestral diversity improves discovery and fine-mapping of genetic loci for anthropometric traits—The Hispanic/Latino Anthropometry Consortium

**DOI:** 10.1016/j.xhgg.2022.100099

**Published:** 2022-03-11

**Authors:** Lindsay Fernández-Rhodes, Mariaelisa Graff, Victoria L. Buchanan, Anne E. Justice, Heather M. Highland, Xiuqing Guo, Wanying Zhu, Hung-Hsin Chen, Kristin L. Young, Kaustubh Adhikari, Nicholette D. Palmer, Jennifer E. Below, Jonathan Bradfield, Alexandre C. Pereira, LáShauntá Glover, Daeeun Kim, Adam G. Lilly, Poojan Shrestha, Alvin G. Thomas, Xinruo Zhang, Minhui Chen, Charleston W.K. Chiang, Sara Pulit, Andrea Horimoto, Jose E. Krieger, Marta Guindo-Martínez, Michael Preuss, Claudia Schumann, Roelof A.J. Smit, Gabriela Torres-Mejía, Victor Acuña-Alonzo, Gabriel Bedoya, Maria-Cátira Bortolini, Samuel Canizales-Quinteros, Carla Gallo, Rolando González-José, Giovanni Poletti, Francisco Rothhammer, Hakon Hakonarson, Robert Igo, Sharon G. Adler, Sudha K. Iyengar, Susanne B. Nicholas, Stephanie M. Gogarten, Carmen R. Isasi, George Papnicolaou, Adrienne M. Stilp, Qibin Qi, Minjung Kho, Jennifer A. Smith, Carl D. Langefeld, Lynne Wagenknecht, Roberta Mckean-Cowdin, Xiaoyi Raymond Gao, Darryl Nousome, David V. Conti, Ye Feng, Matthew A. Allison, Zorayr Arzumanyan, Thomas A. Buchanan, Yii-Der Ida Chen, Pauline M. Genter, Mark O. Goodarzi, Yang Hai, Willa Hsueh, Eli Ipp, Fouad R. Kandeel, Kelvin Lam, Xiaohui Li, Jerry L. Nadler, Leslie J. Raffel, Kathryn Roll, Kevin Sandow, Jingyi Tan, Kent D. Taylor, Anny H. Xiang, Jie Yao, Astride Audirac-Chalifour, Jose de Jesus Peralta Romero, Fernando Hartwig, Bernando Horta, John Blangero, Joanne E. Curran, Ravindranath Duggirala, Donna E. Lehman, Sobha Puppala, Laura Fejerman, Esther M. John, Carlos Aguilar-Salinas, Noël P. Burtt, Jose C. Florez, Humberto García-Ortíz, Clicerio González-Villalpando, Josep Mercader, Lorena Orozco, Teresa Tusié-Luna, Estela Blanco, Sheila Gahagan, Nancy J. Cox, Craig Hanis, Nancy F. Butte, Shelley A. Cole, Anthony G. Comuzzie, V. Saroja Voruganti, Rebecca Rohde, Yujie Wang, Tamar Sofer, Elad Ziv, Struan F.A. Grant, Andres Ruiz-Linares, Jerome I. Rotter, Christopher A. Haiman, Esteban J. Parra, Miguel Cruz, Ruth J.F. Loos, Kari E. North

**Affiliations:** 1 Department of Biobehavioral Health, Pennsylvania State University, 219 Biobehavioral Health Building, University Park, PA 16802, USA; 2 Department of Epidemiology, Gillings School of Global Public Health, University of North Carolina at Chapel Hill, Chapel Hill, NC 27599, USA; 3 Department of Biomedical and Translational Informatics, Geisinger Health System, Danville, PA 17822, USA; 4 The Institute for Translational Genomics and Population Sciences, Department of Pediatrics, The Lundquist Institute for Biomedical Innovation at Harbor-UCLA Medical Center, Torrance, CA 90502 USA; 5 Vanderbilt Genetics Institute, Division of Genetic Medicine, Department of Medicine, Vanderbilt University Medical Center, Nashville, TN 37232, USA; 6 School of Mathematics and Statistics, Faculty of Science, Technology, Engineering and Mathematics, The Open University, MK7 6AA Milton Keynes, UK; 7 Department of Biochemistry, Wake Forest School of Medicine, Winston-Salem, NC 27101, USA; 8 Center for Applied Genomics, Division of Human Genetics, Department of Pediatrics, The Children’s Hospital of Philadelphia, Philadelphia, PA 19104, USA; 9 Laboratory of Genetics and Molecular Cardiology, Heart Institute, University of São Paulo, São Paulo 05508-220, Brazil; 10 Department of Sociology, University of North Carolina at Chapel Hill, Chapel Hill, NC 27599, USA; 11 Carolina Population Center, University of North Carolina at Chapel Hill, Chapel Hill, NC 27599, USA; 12 Division of Pediatric and Public Health, Adams School of Dentistry, University of North Carolina at Chapel Hill, Chapel Hill, NC 27599, USA; 13 Center for Genetic Epidemiology, Department of Preventive Medicine, Keck School of Medicine, University of Southern California, Los Angeles, CA 90033, USA; 14 Department of Quantitative and Computational Biology, University of Southern California, Los Angeles, CA 90007, USA; 15 Vertex Pharmaceuticals, W2 6BD Oxford, UK; 16 The Charles Bronfman Institutes for Personalized Medicine, Icahn School of Medicine at Mount Sinai, New York, NY 10029, USA; 17 The Novo Nordisk Center for Basic Metabolic Research, University of Copenhagen, 2200 Copenhagen, Denmark; 18 Hasso Plattner Institute, University of Potsdam, Digital Health Center, 14482 Potsdam, Germany; 19 Department of Research in Cardiovascular Diseases, Diabetes Mellitus, and Cancer, Population Health Research Center, National Institute of Public Health, Cuernavaca, Morelos 62100, Mexico; 20 National Institute of Anthropology and History, Mexico City 06600, Mexico; 21 Molecular Genetics Investigation Group, University of Antioquia, Medellín 1226, Colombia; 22 Department of Genetics, Federal University of Rio Grande do Sul, Porto Alegre 90040-060, Brazil; 23 Population Genomics Applied to Health Unit, The National Institute of Genomic Medicine and the Faculty of Chemistry at the National Autonomous University of Mexico, Mexico City 04510, Mexico; 24 Laboratorios de Investigación y Desarrollo, Facultad de Ciencias y Filosofía, Universidad Peruana Cayetano Heredia, Lima 15102, Peru; 25 Patagonian Institute of the Social and Human Sciences, Patagonian National Center, Puerto Madryn U9120, Argentina; 26 Institute of High Studies, University of Tarapacá, Arica 1000000, Chile; 27 Department of Population and Quantitative Health Sciences, Case Western Reserve University, Cleveland, OH 44106, USA; 28 Division of Nephrology and Hypertension, Harbor-University of California Los Angeles Medical Center, Torrance, CA 90502, USA; 29 Department of Medicine, David Geffen School of Medicine at University of California, Los Angeles, CA 90095, USA; 30 Department of Biostatistics, University of Washington, Seattle, WA 98195, USA; 31 Department of Epidemiology and Population Health, Albert Einstein College of Medicine, Bronx, NY 10461, USA; 32 National Heart, Lung and Blood Institute, Bethesda, MD 20892, USA; 33 Department of Epidemiology, School of Public Health, University of Michigan, Ann Arbor, MI 48109, USA; 34 Department of Biostatistics and Data Science, Wake Forest School of Medicine, Winston-Salem, NC 27101, USA; 35 Division of Public Health Sciences, Wake Forest School of Medicine, Winston-Salem, NC 27101, USA; 36 Department of Preventive Medicine, Keck School of Medicine, University of Southern California, Los Angeles, CA 90032, USA; 37 Department of Ophthalmology and Visual Sciences, Department of Biomedical Informatics, Division of Human Genetics, The Ohio State University, Columbus, OH 43210, USA; 38 Department of Family Medicine, University of California, San Diego, CA 92161, USA; 39 Department of Medicine, Keck School of Medicine, University of Southern California, Los Angeles, CA 90033, USA; 40 Department of Medicine, Division of Endocrinology, The Lundquist Institute for Biomedical Innovation at Harbor-UCLA Medical Center, Torrance, CA 90502, USA; 41 Division of Endocrinology, Diabetes, and Metabolism, Department of Medicine, Cedars-Sinai Medical Center, Los Angeles, CA 90048, USA; 42 Department of Internal Medicine, The Ohio State University Wexner Medical Center, Columbus, OH 43210, USA; 43 Department of Translational Research & Cellular Therapeutics, Beckman Research Institute of City of Hope, Duarte, CA 91010, USA; 44 Department of Pharmacology at New York Medical College School of Medicine, Valhalla, NY 10595, USA; 45 Division of Genetic and Genomic Medicine, Department of Pediatrics, University of California, Irvine, CA 92697, USA; 46 Research and Evaluation Branch, Kaiser Permanente of Southern California, Pasadena, CA 91101, USA; 47 Medical Research Unit in Biochemistry, Specialty Hospital, National Medical Center of the Twenty-First Century, Mexican Institute of Social Security, Mexico City 06725, Mexico; 48 Postgraduate Program in Epidemiology, Federal University of Pelotas, Pelotas 96010-610, Brazil; 49 Department of Human Genetics and South Texas Diabetes and Obesity Institute, School of Medicine, University of Texas Rio Grande Valley, Brownsville and Edinburg, TX 78520 and 78539, USA; 50 Department of Medicine, School of Medicine, University of Texas Health San Antonio, San Antonio, TX 78229, USA; 51 Department of Internal Medicine, Section of Molecular Medicine, Wake Forest School of Medicine, Winston-Salem, NC 27109, USA; 52 Department of Public Health Sciences, School of Medicine, and the Comprehensive Cancer Center, University of California Davis, Davis, CA 95616, USA; 53 Departments of Epidemiology & Population Health and Medicine-Oncology, Stanford University School of Medicine, Stanford, CA 94305, USA; 54 Division of Nutrition, Salvador Zubirán National Institute of Health Sciences and Nutrition, Mexico City 14080, Mexico; 55 Programs in Metabolism and Medical and Population Genetics, Broad Institute of the Massachusetts Institute of Technology and Harvard, Cambridge, MA 02142, USA; 56 Department of Medicine, Harvard Medical School, Boston, MA 02115, USA; 57 Diabetes Unit and Center for Genomic Medicine, Massachusetts General Hospital, Boston, MA 02114, USA; 58 Laboratory of Immunogenomics and Metabolic Diseases, National Institute of Genomic Medicine, Mexico City 14610, Mexico; 59 Center for Diabetes Studies, Research Unit for Diabetes and Cardiovascular Risk, Center for Population Health Studies, National Institute of Public Health, Mexico City 14080, Mexico; 60 Molecular Biology and Medical Genomics Unity, Institute of Biomedical Research, The National Autonomous University of Mexico and the Salvador Zubirán National Institute of Health Sciences and Nutrition, Mexico City 14080, Mexico; 61 Center for Community Health, Division of Academic General Pediatrics, University of California at San Diego, San Diego, CA 92093, USA; 62 University of Texas Health Science Center at Houston, Houston, TX 77030, USA; 63 United States Department of Agriculture, Agricultural Research Service, The Children’s Nutrition Research Center, and the Department Pediatrics, Baylor College of Medicine, Houston, TX 77030, USA; 64 Population Health Program, Texas Biomedical Research Institute, San Antonio, TX 78227, USA; 65 The Obesity Society, Silver Spring, MD 20910, USA; 66 Department of Nutrition and Nutrition Research Institute, University of North Carolina at Chapel Hill, Kannapolis, NC 28081, USA; 67 Division of Sleep and Circadian Disorders, Brigham and Women’s Hospital, Boston, MA 02115, USA; 68 Division of General Internal Medicine, Department of Medicine, Helen Diller Family Comprehensive Cancer Center, Institute for Human Genetics, University of California, San Francisco, San Francisco, CA 94115, USA; 69 Ministry of Education Key Laboratory of Contemporary Anthropology and Collaborative Innovation Center of Genetics and Development, School of Life Sciences and Human Phenome Institute, Fudan University, Shanghai 200438, China; 70 Department of Genetics, Evolution and Environment, and Genetics Institute of the University College London, London WC1E 6BT, UK; 71 Laboratory of Biocultural Anthropology, Law, Ethics, and Health, Aix-Marseille University, Marseille 13385, France; 72 Department of Anthropology, University of Toronto- Mississauga, Mississauga, ON L5L 1C6, Canada; 73 Novo Nordisk Foundation Center for Basic Metabolic Research, Faculty of Health and Medical Sciences, University of Copenhagen, Copenhagen, Denmark; 74 Carolina Center for Genome Sciences, University of North Carolina at Chapel Hill, Chapel Hill, NC 27514, USA

**Keywords:** Hispanic/Latino, anthropometrics, obesity, diversity, trans-ancestral or trans-ethnic, fine-mapping, population stratification

## Abstract

Hispanic/Latinos have been underrepresented in genome-wide association studies (GWAS) for anthropometric traits despite their notable anthropometric variability, ancestry proportions, and high burden of growth stunting and overweight/obesity. To address this knowledge gap, we analyzed densely imputed genetic data in a sample of Hispanic/Latino adults to identify and fine-map genetic variants associated with body mass index (BMI), height, and BMI-adjusted waist-to-hip ratio (WHRadjBMI). We conducted a GWAS of 18 studies/consortia as part of the Hispanic/Latino Anthropometry (HISLA) Consortium (stage 1, n = 59,771) and generalized our findings in 9 additional studies (stage 2, n = 10,538). We conducted a trans-ancestral GWAS with summary statistics from HISLA stage 1 and existing consortia of European and African ancestries. In our HISLA stage 1 + 2 analyses, we discovered one BMI locus, as well as two BMI signals and another height signal each within established anthropometric loci. In our trans-ancestral meta-analysis, we discovered three BMI loci, one height locus, and one WHRadjBMI locus. We also identified 3 secondary signals for BMI, 28 for height, and 2 for WHRadjBMI in established loci. We show that 336 known BMI, 1,177 known height, and 143 known WHRadjBMI (combined) SNPs demonstrated suggestive transferability (nominal significance and effect estimate directional consistency) in Hispanic/Latino adults. Of these, 36 BMI, 124 height, and 11 WHRadjBMI SNPs were significant after trait-specific Bonferroni correction. Trans-ancestral meta-analysis of the three ancestries showed a small-to-moderate impact of uncorrected population stratification on the resulting effect size estimates. Our findings demonstrate that future studies may also benefit from leveraging diverse ancestries and differences in linkage disequilibrium patterns to discover novel loci and additional signals with less residual population stratification.

## Teaser

Large-scale genetic analyses of diverse populations hold great potential for advancing the field. In our sample of Hispanic/Latino adults, and when combined with publicly available results from other ancestries, we identified, described, and characterized 42 novel genomic findings. We additionally illustrated that novel biologic insights can be garnered from studying ancestrally diverse populations. Overall, our findings indicate the added value of building large, more diverse genome-wide association studies in the future.

## Introduction

A complex interplay between political, social, and economic factors has led to an increasing obesogenic global environment in which many low-to-middle income nations have experienced a rapid transition from under-nutrition and growth stunting to over-nutrition and obesity.^1^ In Latin America, by 2016, 35% of the total population was overweight (body mass index [BMI] 25 to <30 kg/m^2^) and another 23% was living with obesity (BMI ≥ 30 kg/m^2^).^2^ In Mexico, it is projected that by 2050 only 12% of men and 9% of women will have a healthy weight (BMI < 25 kg/m^2^).^3^ In South America in 2010–2011, the prevalence of obesity was 36%, but abdominal obesity (based on waist circumference) was even more common (53%).^4^

Ancestry may also play a role in anthropometric-related health disparities in Hispanic/Latino populations. Previous studies have described the historical contexts leading to admixture in Latin American populations^5^^,^^6^ as characterized by highly diverse (variable) ancestral proportions^7^, ^8^, ^9^ from any of the following regions: the Americas, Europe, Africa, and East Asia.^10^, ^11^, ^12^, ^13^, ^14^, ^15^ The proportion of Native American ancestry is associated with obesity-related traits, and even more strongly associated with height.^16^^,^^17^ Height is inversely associated with proportion of Native American ancestry, even after taking into account that over time populations globally have become taller due to mainly non-genetic nutritional factors.^16^ The ultimate drivers of this association remain unclear; it is possible that genetic factors and/or socio-economic factors strongly associated with Native American ancestry could be responsible. Recent studies are starting to provide relevant insights into this topic, including a recent genome-wide association study (GWAS) in Peru^18^ that identified a missense variant in the *FBN1* gene (rs200342067) that has the largest effect size so far described for common height-associated variants in human populations. In the 1000 Genomes Project samples, rs200342067 is only present in two Latin American samples (MXL, 0.78%; and PEL, 4.12%), and yet the authors reported that this missense variant shows subtle evidence of positive selection in the Peruvian population.^18^

In the US, as in other high-income nations, both the population size and diversity in national origins (backgrounds) of Hispanic/Latinos have been increasing over the past several decades,^19^ with 24% of the US adult population identifying as Hispanic/Latino by 2065.^19^ US Hispanic/Latino adults and children/adolescents face a greater burden of obesity than their non-Hispanic white counterparts.^20^, ^21^, ^22^, ^23^

Thus, there is a need to study Hispanic/Latino populations to fully address these disparities.^23^^,^^24^ Specifically, we sought to understand the role that Native American or other under-studied components of admixture have on the genetic architecture of anthropometric traits in Hispanic/Latinos, and their relationship with gene expression. To date, no large-scale GWAS of anthropometric traits has been conducted among Hispanic/Latino populations; we therefore performed a large-scale genomic study of multiple anthropometric traits, including BMI, height, and waist-to-hip ratio adjusted for BMI (WHRadjBMI), in Hispanic/Latino populations to describe what may be novel loci, or new signals in established loci, for this population.

## Materials and methods

### Hispanic/Latino study samples

The Hispanic/Latino Anthropometry (HISLA) Consortium is comprised of 27 studies/consortia of adult participants. First, HISLA stage 1 includes 17 studies and one consortium (Consortium for the Analysis of the Diversity and Evolution of Latin America [CANDELA]^17^) collectively representing up to 59,771 adults, depending on the trait, from Brazil, Chile, Colombia, Mexico, Peru, or the US with self-reported heritage from across Spanish-speaking Latin America, or Native American heritage, primarily Pima and Zuni^25^ (Table S1). HISLA stage 2 includes 9 studies with up to 10,538 adults from across Spanish-speaking Latin America or with related heritage and living in the US (Table S1).

This study was approved by the institutional review boards of the University of North Carolina at Chapel Hill, and all contributing studies had received prior institutional review boards approval for each study’s activities.

### Anthropometric traits

BMI is a commonly derived index of obesity risk and is calculated as the ratio of body weight to height squared (kg/m^2^). Adult height was measured or self-reported using either metric or US units and then converted to meters. Waist-to-hip ratio (WHR) is used to capture central fat deposition and is derived from the circumference of the waist at the umbilicus compared with the circumference of the hip at the maximum protrusion of the gluteal muscles.

Residuals were calculated by sex and/or case status, adjusting for age, age^2^, and study-specific covariates (e.g., center; principal components [PCs]). For WHR, we also adjusted for BMI when creating the residuals to isolate the central deposition of fat from overall body mass. Residuals were then used to create inverse normalizations of BMI and WHRadjBMI, and *Z* scores of height (=residual/standard deviation for all residuals). In family-based studies, the residuals were calculated in women and men together, adjusting for age, sex, and other study covariates including PCs. Descriptive statistics on the covariates and anthropometric measures are provided for each study’s analytic sample in Table S2. Only one family-based study in stage 1 and two non-family-based studies in stage 2 (Genetics of Latinos Diabetic Retinopathy, 0.3% <18 years; and Mapping the Genes for Hypertension, Insulin Resistance, and Salt Sensitivity Study, 3.9%) included a small subset of adolescents aged 15–17 years, each less than 5% of the total sample. All other study samples included individuals aged 18–98 years.

### Childhood/adolescence study samples, anthropometric traits, and obesity

We assembled an independent sample of children/adolescents with anthropometrics from three studies from the US, Mexico, and Chile (Table S3). The distribution of covariates and anthropometrics of the samples of children/adolescents in each analysis are described in Table S4. First, childhood/adolescent obesity was defined as ≥95th BMI-for-age percentile (versus ≤50th BMI-for-age percentile), based on the Centers for Disease Control and Prevention growth curves,^26^ as done in previous analyses of childhood obesity.^27^ We used these two analyses to look up the BMI and height findings from our adult HISLA meta-analysis as well as our trans-ancestral analyses. This resulted in 1,814 children/adolescents aged 2–18 years in a case-control analysis of childhood obesity (Tables S3 and S4). Second, BMI and height-for-age *Z* scores were calculated in children/adolescents aged 5–18 years from the US and Chile (Table S4) based on the more international reference growth curves from the World Health Organization.^28^ In Viva la Familia, a family-based study,^29^ these residuals were calculated adjusting for sex in the combined sample. The resulting BMI and height-for-age *Z* scores were available for 1,914 and 1,945 children/adolescents, respectively.

### SNP imputation and statistical analyses

We generated autosomal genome-wide imputed data based on 1000 Genomes phase 1 and 3 references, except for two studies that contributed Exomechip and MetaboChip (Illumina, San Diego, CA) genotypes and one study that blended genotypes from multiple platforms (Tables S5 and S6). Principal-component analyses (PCA) were conducted in each study (see select examples provided in Figures S1–S3) to capture the main components of genetic ancestry from the Americas, Europe, Africa, and East Asia. Studies with samples from related individuals accommodated this non-independence by projecting their PCA from the reference to the study sample, and by accounting for relatedness using either generalized estimating equations^30^ or mixed linear models.^9^^,^^31^ Assuming an additive genetic model, we tested for the association of over 20 million autosomal variants on our traits, accounting for all trait- or study-specific covariates (e.g., center, PCs).

### Meta-analyses of HISLA stage 1 + 2

The studies of the HISLA Consortium were meta-analyzed in two stages: discovery (stage 1) and replication (stage 2). Stage 1 included a total sample of 59,771 individuals with data on BMI, 56,161 with height, and 42,455 with WHRadjBMI. All stage 1 studies/consortia provided full genome-wide analysis results. All SNPs that met our significance criteria were brought forward for replication in stage 2, which included 10,538 individuals with data on BMI, 8,110 with height, and 4,393 with WHRadjBMI. All reported association results passed our quality control criteria; i.e., variants with low quality (info score < 0.4 or R^2^ < 0.3), minor allele count (MAC) < 5, or sample size < 100 were removed. We meta-analyzed effects across all studies using a fixed-effect inverse variance weighted meta-analysis with genomic control in METAL.^32^ Given the unique patterns of admixture and ancestry represented by the Brazilian or Native American samples, we conducted sensitivity analyses in stage 1 studies (i.e., comparing the inclusion and exclusion of the Baependi Heart Study, the 1982 Pelotas Birth Cohort Study, and the Family Investigation of Nephropathy and Diabetes substudy of individuals of Pima and Zuni heritage) to assess the influence of these three studies on the meta-analysis results. CANDELA was retained in all analyses as <10% of the consortium’s samples came from Brazil, primarily originating from the South of Brazil and being characterized as having high European heritage and less Native American or African admixture.^17^ We provide the quantile-quantile plots for all analyses in Figure S4.

Regional plots of all GWAS significant HISLA stage 1 findings were plotted using LocusZoom. From stage 1, we selected lead variants for replication that met genome-wide significance (p < 5 × 10^−8^) that were independent of each other. In cases where stage 2 studies did not have the lead variant, we selected two proxies per lead variant with a linkage disequilibrium (LD) r^2^ ≥ 0.9 using 1000 Genomes AMR. Stage 2 studies provided a list of the requested lead variants and/or their proxies from stage 1 for replication. Stage 2 studies were meta-analyzed and subsequently combined with stage 1 using METAL.^25^ Effect heterogeneity was assessed through I^2^ across all 27 HISLA adult studies/consortia by entering each study separately into the meta-analysis, irrespective of stage. The characteristics of the final SNP array data used in the HISLA adult studies and the children/adolescent Hispanic/Latino studies are summarized separately in Tables S5 and S6.

### Meta-analyses of HISLA stage 1 with other ancestral consortia

In addition to a Hispanic/Latino-only meta-analysis, we combined the HISLA stage 1 meta-analysis with data from previous large-scale GWAS meta-analyses of European (the Genetic Investigation of Anthropometric Traits [GIANT] Consortium,^33^, ^34^, ^35^ N ∼ 300,000) and/or African (the African Ancestry Anthropometry Genetics Consortium [AAAGC],^36^^,^^37^ N ∼ 50,000) descent populations. We used fixed-effect inverse variance weighted meta-analytic techniques in METAL to generate our trans-ancestral meta-analysis.^32^ We then assessed (1) the transferability of the findings from the BMI, height,^38^ and WHRadjBMI^39^ trans-ancestral meta-analyses to an independent sample of Hispanic/Latino children/adolescents or (2) the replication of the signal in the British subsample GWAS of the United Kingdom Biobank (UKBB). LD plots and regional plots are shown in the supplemental information (Figures S5–S53).

### Thresholds for conditional signals, discovery, and transferability

We conducted approximate conditional analyses using genome-wide complex trait analysis (version 1.93.1) software. For HISLA analyses, we used our stage 1 discovery results with the Hispanic Community Health Study/Study of Latinos (HCHS/SOL) as the LD reference dataset. For the approximate conditional trans-ancestral analyses, we used our trans-ancestral results from HISLA stage 1, AAAGC, or GIANT and a trans-ancestral LD reference dataset of Europeans and African Americans from the Atherosclerosis Risk in Communities (ARIC) cohort, and Hispanic/Latinos from the HCHS/SOL cohort, as a representation of the ancestry distribution of our meta-analysis. In both conditional analyses (HISLA-only and trans-ancestral results), we first identified all independent SNPs using the --cojo-slct command. Then, we conditioned each of these independent SNPs on all known SNPs from GWAS (curated in the GWAS catalog) or reported as part of targeted-array analyses published through December 2019 (BMI,^33^^,^^36^^,^^38^^,^^40^, ^41^, ^42^, ^43^, ^44^, ^45^, ^46^, ^47^, ^48^, ^49^, ^50^, ^51^, ^52^, ^53^, ^54^, ^55^ height,^34^^,^^38^^,^^47^^,^^50^^,^^55^^,^^56^ and WHRadjBMI^35^^,^^36^^,^^39^^,^^44^^,^^46^^,^^54^^,^^55^^,^^57^, ^58^, ^59^, ^60^, ^61^) within 10 Mb (±5 Mb) of the lead SNP. The trans-ancestral meta-analysis results with a p < 5 × 10^−8^ after conditioning on known SNPs were taken forward for replication in the British subsample of the UKBB.

In this paper, we consider a signal as replicated when the effect of an allele is observed in two independent populations with the same or overlapping ancestral background, whereas generalization (transferability) refers to the observation of the same signal in an independent sample but with a distinct ancestral background, or distinct period of the life course. Furthermore, SNP associations were then defined as either newly discovered or established, depending on their location. An established locus was defined as an SNP association within ±500 kb of at least one previously identified index SNP, otherwise the association was considered a newly discovered locus.

We designated our Hispanic/Latino SNP associations within either newly discovered or established loci as “novel” if they met the following criteria for replication: (1) were associated at p < 5 × 10^−8^ in HISLA stage 1 and directionally consistent in the stage 2 independent sample, and (2) the addition of stage 2 samples improved the estimated p value of the stage 1 + 2 meta-analysis. For the trans-ancestral analyses, the designation of a signal as novel was based on SNPs that were: (1) associated at p < 5 × 10^−8^ in the combined HISLA, AAAGC, and GIANT meta-analysis, and (2) directionally consistent with the trans-ancestral meta-analysis and associated at p < 5 × 10^−2^ in an independent sample of Hispanic/Latino children/adolescents (generalized across age period) or in the British subsample GWAS from the UKBB (replication).

Hispanic/Latino SNP effects were considered to transfer (or generalize) to Hispanic/Latino children/adolescents or to African or European ancestry adults if they were: (1) directionally consistent, (2) associated at p < 5 × 10^−2^, and (3) had a heterogeneity of I^2^ < 75% in the Hispanic/Latino children/adolescent lookups, the adult AAAGC, or the adult GIANT GWAS lookups. SNP effects of variants previously associated with anthropometric traits in non-Hispanic/Latino populations (i.e., index published SNPs) were considered to be transferable (generalizable) to Hispanic/Latinos only if they were: (1) directionally consistent, (2) displayed a p < 5 × 10^−2^, and (3) had little to moderate effect heterogeneity (I^2^ < 75%) in stage 1.

### Fine-mapping methods

We used FINEMAP^62^ for analyses of the newly discovered loci identified as part of the HISLA stage 1 meta-analysis or trans-ancestral meta-analysis, for both established and novel loci. For the established loci, we included index SNP associations published as of April 2018 (BMI,^33^^,^^36^^,^^40^^,^^42^, ^43^, ^44^^,^^46^^,^^48^^,^^51^, ^52^, ^53^, ^54^ height,^34^^,^^50^^,^^56^ and WHRadjBMI^35^^,^^36^^,^^44^^,^^46^^,^^59^) prior to the publications with the UKBB results.^38^^,^^39^ We used a 1 Mb region subset of the summary statistics from the stage 1 meta-analyses and HCHS/SOL^9^ unrelated sample set (N ∼ 7,670) to calculate the LD for each locus. For consistency with the FINEMAP package, we refer to the results using similar language; however, we do not mean to imply that the SNP(s) in the “causal set” are causative variants.

For trans-ancestral fine-mapping of the novel loci or new signals identified in the trans-ancestral meta-analysis of HISLA, AAAGC, and GIANT, we used a 1 Mb region defining each locus using the summary statistics of the given meta-analysis. We calculated the LD for Hispanic/Latino samples using the HCHS/SOL^9^ unrelated sample (N ∼ 7,670). For African and European ancestry samples, we calculated the LD using the ARIC unrelated sample that included self-reported African ancestry (N ∼ 2,800) and European ancestry (N ∼ 9,700). We weighted the LD matrices by the GWAS sample sizes for each trait (HISLA range, ∼42,400–56,100; AAAGC, 20,300–42,700; GIANT, 210,000–330,000).

All regions allowed up to a maximum of 10 causal variants, as defined by FINEMAP. The cumulative 95th percentile credible set was calculated from the estimated posterior probabilities. Convergence failed for three regions (lead SNPs at known height loci: rs2902635, rs6900530, and rs4425978) using the stochastic approach. For these three regions, we used the conditional approach to determine number of causal variants.

### Gene expression and other bioinformatic analyses

We performed association analyses of measured whole blood gene expression in 606 individuals from the Cameron County Hispanic Cohort.^63^ RNA sequencing was conducted using 150 bp paired-end reads on the Illumina NovaSeq 6000 by Vanderbilt Technologies for Advanced Genomics. Initial sequencing quality was checked by FastQC.^64^ STAR-2.7.8a was applied to align sequencing reads alignment to the human genome reference (UCSC, hg38),^65^ and the aligned reads were assigned to genes using featureCounts.^66^ We excluded either samples with less than 15 million total aligned reads, a rate of successful alignment of less than 20%, or less than 15 million total assigned reads. The sequencing library size was normalized using DESeq2^67^ and read counts were transformed using variance stabilizing transformations (vst in DESeq2 package). We performed expression quantitative trait loci (eQTL) analysis with our top HISLA SNP findings, by modeling SNP dosages (exposure) in a linear regression of gene expression levels (outcomes), for each gene within the 1 Mb interval around each lead SNP. We inverse normalized the gene expression levels and adjusted for age, sex, and three PCs to capture population substructure. Bonferroni correction for each region varied according to the number of SNPs tested.

To gain further insight into the possible functional role of the identified variants and to assess their relevance to other phenotypes, we conducted bioinformatic queries of our potentially novel loci and new signals within known loci in multiple publicly available databases, including PhenoScanner,^68^ RegulomeDB,^69^ Haploreg,^70^ UCSC GenomeBrowser,^71^ and GTEx.^72^

### Trans-ancestral findings to account for population structure in previous GWAS

We demonstrated the degree to which the present trans-ancestral meta-analysis could lessen the bias induced by population stratification, using height from HISLA as an example. We first conducted PCA on the four European populations (CEU, GBR, IBS, and TSI) from 1000 Genomes. We excluded the Finnish population because of its known unique demographic history that could drive or dominate the top PCs in a limited sample.^33^ We only used biallelic SNPs with minor allele frequency (MAF) > 5% in the four European populations, and then pruned them by both distance and LD using PLINK 1.9.^73^ Specifically, we pruned the dataset such that no two SNPs were closer than 2 kb, and then pruned using a 50 SNP LD window (moving in steps of 5 SNPs), such that no SNPs had r^2^ > 0.2. We further removed SNPs in regions of long-range LD.^74^ PCA was performed on the remaining SNPs using Eigensoft version 7.2.1.

We performed linear regressions of individual PC values on the allelic genotype count for each polymorphic variant in the four European populations from 1000 Genomes and used the resulting regression coefficients as the estimate of the variant’s PC loading. For each PC, we then computed Pearson correlation coefficients of PC loadings and effect sizes (of variants with MAF > 1%) from each GWAS summary statistic. We estimated p values based on Jackknife standard errors, by splitting the genome into 1,000 blocks with an equal number of variants. If the GWAS summary statistics are not biased by residual stratification (in this case due to European geographical structure), the correlation coefficients would be expected to be zero. If there was significant correlation in either the GIANT dataset or the HISLA stage 1, AAAGC, and GIANT trans-ancestral meta-analysis, we then further evaluated the improvement of bias due to stratification in trans-ancestral meta-analysis by comparing the correlation coefficients in the trans-ancestral meta-analysis with those in GIANT. Restricting to variants shared between GIANT and the trans-ancestral meta-analysis, we computed their difference in correlation coefficients of PC loadings and effect sizes, and estimated p values again based on Jackknife standard errors from 1,000 equal sized blocks.

## Results

### Discovery of one BMI locus in Hispanic/Latino adults

**Figure 1:**
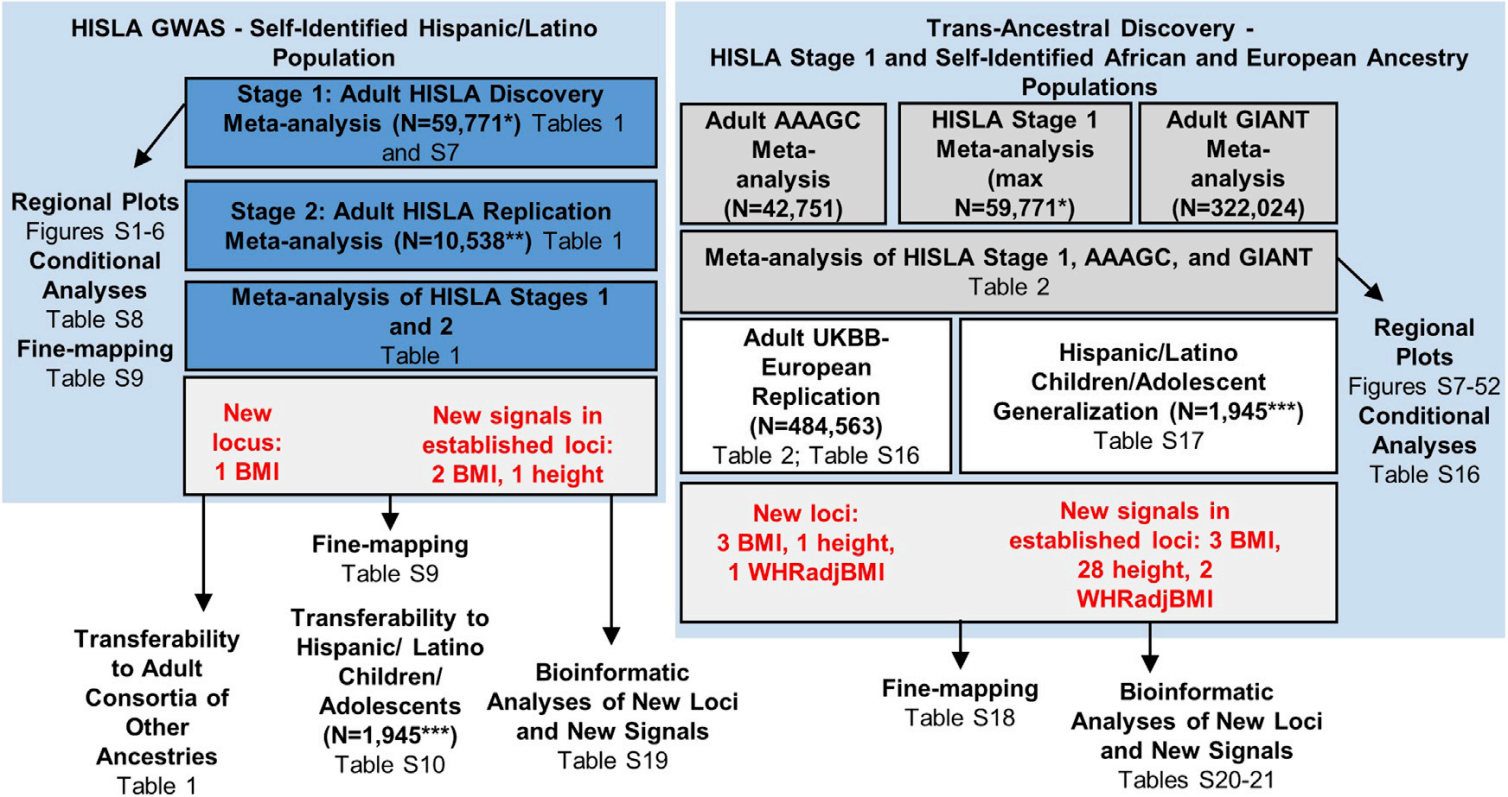
Flowchart of the design and discovery of 6 loci and 36 signals in known loci in the HISLA meta-analysis and the trans-ancestral meta-analysis of HISLA and consortia of other ancestries ∗Stage 1 maximum sample sizes varied from 59,771 for BMI, 56,161 for height, to 42,455 for WHRadjBMI (sex combined). ∗∗Stage 2 sample sizes varied from 10,538 for BMI, 8,110 for height, to 4,393 for WHRadjBMI (sex combined). Actual sample sizes may vary by SNP. ∗∗∗The BMI and height-for-age *Z* score models were conducted using up to 1,914 and 1,945 of children/adolescents, respectively. In contrast, the obesity case-control study compared up to 1,814 children/adolescents who were ≥95th versus ≤50th BMI-for-age percentiles.

The first goal of this study was to conduct a genome-wide meta-analysis of anthropometric traits in Hispanic/Latino adults to identify loci in an under-studied population (Figure 1). All regional plots of all potentially novel GWAS significant HISLA stage 1 findings are shown in the supplemental information (Figures S6–S11).

**Figure 2:**
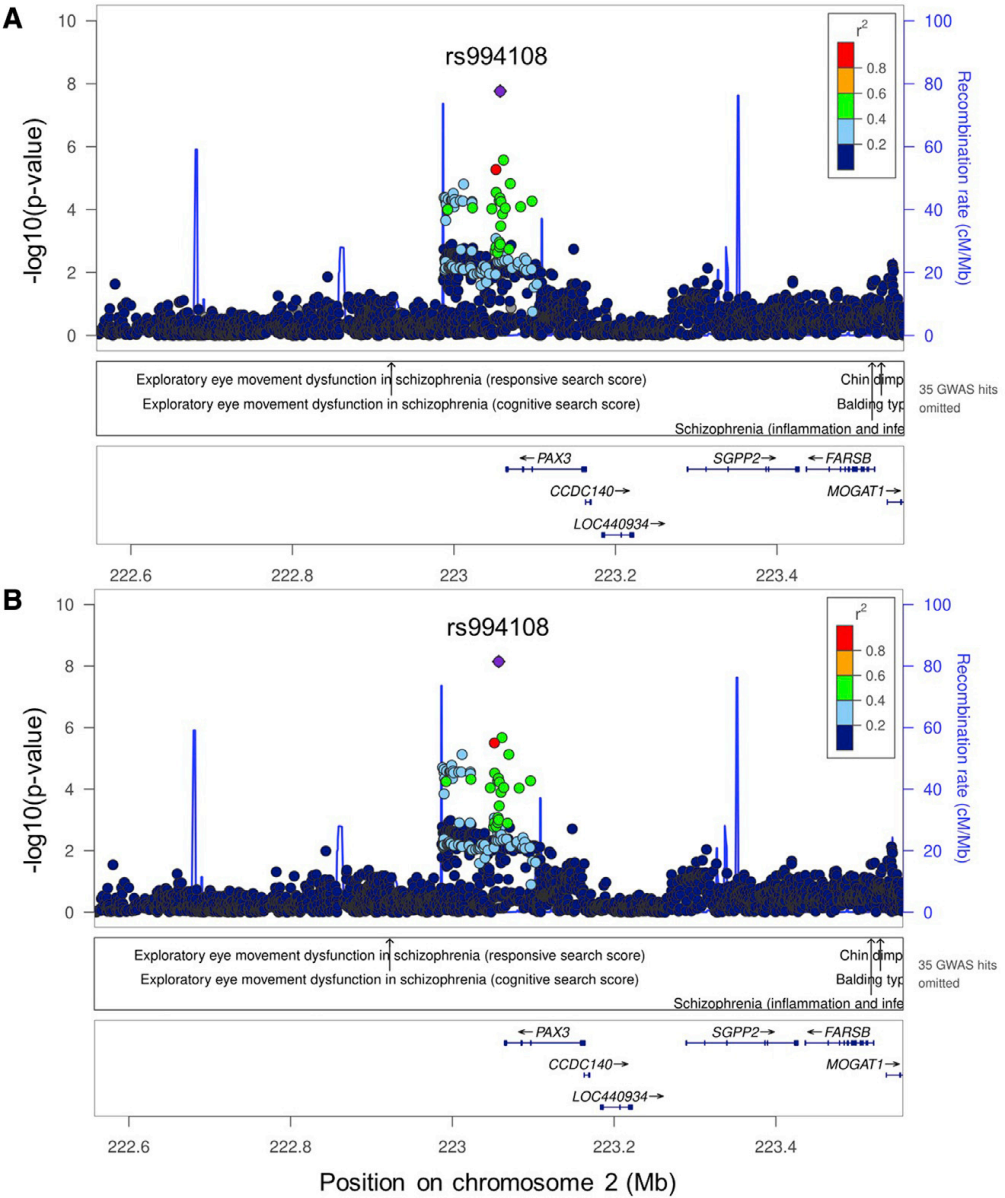
Regional plot of novel body mass index signal at *PAX3* Regional plot, unconditioned (A) and conditioned (B) on established variants within ±500 kb of the lead variant, at the BMI locus at *PAX3* in the HISLA (after excluding Brazilian and Native American samples). Linkage disequilibrium patterns are based on rs994108 (shown by the purple diamond) from the Hispanic Communities in Health Study/Study of Latinos.

No novel anthropometric loci were identified in all HISLA stage 1 samples combined. Yet, when we excluded the samples of exclusively Brazilian or Native American heritage from stage 1, we discovered one locus for adult BMI at *PAX3* on chromosome 2 in the HISLA stage 1 sample (Table S7) and replicated this locus in HISLA stage 2 (Table 1). The lead SNP at this locus, rs994108, is in moderate LD with a previously-reported SNP (rs7559271, r^2^ = 0.46 in 1000 Genomes AMR) (Figure 2) and lies on the same haplotype as reported to influence facial morphology, including position of the nasion (the deepest point on the nasal bridge where the nose meets the forehead) in Europeans^75^ and Hispanic/Latino^76^ descent individuals. Other *PAX3* variants in lower LD with the lead SNP have also been associated with nasion position,^77^ monobrow, and male-pattern baldness.^78^^,^^79^ *PAX3* is a well-known transcription factor in normal embryonic neural crest development and differentiation.^80^ Neural crest cells can give rise to mesenchymal stem cells,^81^ which can in turn give rise to adipocytes;^81^, ^82^, ^83^ thus, the possible role of *PAX3* in adipogenesis may at least partially explain the association signal with BMI near this gene.

**Table 1:** Potentially novel loci and new signals in known loci from the stage 1: adult HISLA discovery; combined with the stage 2: adult HISLA replication sample, and lookups of results from the AAAGC and GIANT consortia

Trait	Locus name	SNP RSID	Genomic region^b^	Chr	Position (hg19)	Effect/other alleles	Stage	EAF	Beta	SE	p value	HetISq	N	Novel? Yes/no^c^
**Novel loci**														

BMI	*PAX3*^a^	rs994108	intergenic	2	223057288	C/A	stage 1: discovery	0.390	0.041	0.007	1.62 × 10^−8^	0	43,048	yes
							stage 2: validation	0.394	0.030	0.016	5.65 × 10^−2^	8.0	9,336	
							stage 1 + 2	0.389	0.038	0.006	2.19 × 10^−9^	0	52,384	
							AAAGC	0.526	0.007	0.007	3.26 × 10^−1^	0	42,751	
							GIANT	0.342	<0.001	0.004	9.81 × 10^−1^	–	233,955	
	*ARRDC3*	rs1505851	intronic	5	90893954	T/C	stage 1: discovery	0.741	0.041	0.007	2.287 × 10^−8^	0	52,365	no
							stage 2: validation	0.709	0.005	0.017	7.62 × 10^−1^	33.3	9,336	
							stage 1 + 2	0.735	0.035	0.007	1.16 × 10^−7^	14.1	61,701	
							AAAGC	0.307	0.027	0.008	7.00 × 10^−4^	46.6	42,752	
							GIANT	0.680	0.001	0.004	7.90 × 10^−1^	–	233,999	
WHRadjBMI (women only)	*DOCK2*^a^	rs6879439	intronic	5	169314869	C/T	stage 1: discovery	0.520	0.060	0.010	1.02 × 10^−8^	0	18,591	no
							stage 2: validation	0.526	0.013	0.028	6.54 × 10^−1^	28.7	2,747	
							stage 1 + 2	0.515	0.049	0.0093	1.57 × 10^−7^	1.9	23,382	
							AAAGC	0.440	0.012	0.012	3.09 × 10^−1^	0	15,600	
							GIANT	0.610	0.003	0.005	6.30 × 10^−1^	–	86,317	
WHRadjBMI (sex combined)	*TAOK3*	rs115981023	intronic	12	118751105	A/G	stage 1: discovery	0.009	0.328	0.057	1.08 × 10^−8^	44.8	19,640	no
							stage 2: validation	0.004	−0.339	0.687	6.22 × 10^−1^	0	1,340	
							stage 1 + 2	0.009	0.308	0.057	5.18 × 10^−8^	52.0	20,980	
							AAAGC	0.050	0.027	0.027	3.07 × 10^−1^	0	15,601	
							GIANT	0.002	no proxy					

**New signals in known loci**														

BMI	*ADCY5*^a^	rs17361324	intronic	3	123131254	T/C	stage 1: discovery	0.280	0.042	0.008	2.60 × 10^−8^	0	43,333	yes
							stage 2: validation	0.269	0.035	0.018	4.70 × 10^−2^	0	9,035	
							stage 1 + 2	0.278	0.041	0.007	2.84 × 10^−9^	0	52,368	
							AAAGC	0.119	0.023	0.011	3.85 × 10^−2^	0	42,682	
							GIANT	0.253	0.013	0.004	9.90 × 10^−4^	–	320,704	
	*ILRUN*	rs148899910	intergenic	6	34232259	C/G	stage 1: discovery	0.275	0.040	0.007	9.03 × 10^−9^	0	54,105	yes
							stage 2: validation	0.282	0.049	0.017	4.43 × 10^−3^	0	9,035	
							stage 1 + 2	0.276	0.041	0.006	1.24 × 10^10^	0	63,140	
							AAAGC	0.316	−0.016	0.008	5.02 × 10^−2^	30.0	42,750	
							GIANT^d^	0.017	0.036	0.012	3.99 × 10^−3^	–	216,522	
Height	*B4GALNT3*	rs215226	intronic	12	591300	A/G	stage 1: discovery	0.550	−0.032	0.005	5.53 × 10^−9^	22.1	52,156	yes
							stage 2: validation	0.565	−0.020	0.017	2.37 × 10^−1^	19.3	6,906	
							stage 1 + 2	0.551	−0.031	0.005	1.98 × 10^−9^	21.8	59,062	
							AAAGC	0.772	−0.031	0.009	8.99 × 10^−4^	24.0	41,327	
							GIANT	0.633	0.006	0.004	1.10 × 10^−1^	–	220,370	

Chr, chromosome; EAF, effect allele frequency; HetIsq, heterogeneity I^2^; N, sample size; WHRadjBMI, waist-to-hip ratio adjusted for BMI; AAAGC, African American Anthropometry Genetics Consortium; GIANT, Genetic Investigation of Anthropometric Traits Consortium.

All studies were meta-analyzed using METAL,^32^ with each study entered individually into the stage 1 + 2 meta-analysis.

a These BMI and WHRadjBMI analyses did not include Brazilian and/or Native American samples.

b Human Genome Organisation-approved gene names.

c New loci or signals are those that were replicated by HISLA stage 2 results that are directionally consistent with stage 1 and remained genome-wide significant after meta-analysis with stage 1.

d Proxy GIANT, rs1573905 (r^2^ = 0.96 AMR).

Another BMI SNP (rs1505851-T) near *ARRDC3* on chromosome 5 associated at genome-wide significance in HISLA stage 1 (Table S7; Figure S6), but did not replicate in stage 2 (MAF = 71%) or generalize to AAAGC or GIANT (effect allele frequency = 31% or 68%; Table 1). However, the association was directionally consistent and showed some signal in AAAGC (p = 7 × 10^−4^). LD patterns at *ARRDC3* appear to be similar across the three ancestries (albeit with a smaller LD block for 1000 Genomes AFR), meaning that lack of generalization may be related to frequency differences, haplotype effects, or a false positive (Figure S5A).

We identified two WHRadjBMI loci at *DOCK2* and *TAOK3* at genome-wide significance in HISLA stage 1 after excluding the Brazilian and Native American samples (Table S7; Figures S7 and S8), yet neither met the p value threshold for replication in HISLA stage 2. The *DOCK2* association for WHRadjBMI observed among women only in stage 1 (Figure S7) was directionally consistent in the female stage 2 sample. There were more SNPs in high LD with rs6879439 in 1000 Genomes AMR (0.8 ≤ r^2^ ≤ 1) than for AFR and EUR references (Figure S5B), which may explain why this SNP association did not generalize to AAAGC or GIANT (Table 1).

The genome-wide significant stage 1 *TAOK3* association was led by a low frequency variant (rs115981023-A, MAF = 0.9%), but the associations at this variant were not directionally consistent across stages (Table 1; Figure S8). In fact, patterns of very high LD were seen with rs115981023 in 1000 Genomes AMR and AFR (Figure S5C), even though this variant is seen in African ancestry at a higher frequency (e.g., MAF = 0.9% in HISLA versus 5% in AAAGC). Similarly, rs115981023 exhibited moderate heterogeneity across stage 1 samples after excluding Brazilian and Native American samples (I^2^ = 45%); evidence of moderate heterogeneity remained (I^2^ = 52%) in the combined meta-analysis of HISLA stage 1 and 2 samples (Table 1). Finally, this variant was the least frequent in European ancestry (MAF = 0.2% in GIANT), which explained the lack of a proxy for generalization in GIANT (Table 1).

No potentially novel loci were identified for height in HISLA stage 1, and the exclusion of the Brazilian and Native American samples did not reveal additional height or WHRadjBMI loci.

### Discovery of three signals in established loci for BMI and height in Hispanic/Latino adults

At two established loci for BMI, we identified additional signals at *ADCY5* and near *ILRUN* (Table S7). These signals were both independent of any previously published anthropometric findings (Table S8; Figures S9 and S10). We replicated these signals in stage 2 with directional consistency and in the combined stage 1 + 2 meta-analysis at GWAS significance (Table 1). We also identified one additional signal for height in an established height locus, *B4GALNT3*, which was independent of the previously reported SNPs for height (Tables S7 and S8; Figure S11). We replicated this signal in stage 2 with directional consistency and a stage 1 + 2 meta-analysis that was GWAS significant (Table 1). In additional gene expression and bioinformatics analyses (Tables S18–S20), we found that each of the three additional signals in established anthropometric loci is supported by an eQTL in whole blood in Hispanic/Latino populations (Table S18), and an eQTL in other relevant tissues, e.g., thyroid, esophagus, artery, using publicly available (non-Hispanic/Latino) datasets (Tables S19 and S20).

### Fine-mapping of Hispanic/Latino anthropometric findings

We fine-mapped the *PAX3* locus for BMI and the three additional signals in known loci (BMI, *ADCY5* and *ILRUN*; height, *B4GALNT3*; Table S9). For the three BMI loci, FINEMAP revealed one potential causal set for each locus at *PAX3*, *ADCY5*, and *ILRUN* loci. For the *PAX3* locus, this 95th percentile credible set contained only nine plausibly causal SNPs, with the lead SNP rs994108 having a very high posterior probability of being causal (0.89, Table S21). However, functional annotation of this SNP was unremarkable (Tables S22 and S23). In contrast, for *ADCY5* and *ILRUN*, FINEMAP revealed one causal configuration for each locus but with much greater uncertainty of the likely functional variant given the size of the credible sets, which contained 14 and 22 SNPs in the credible region for *ADCY5* and *ILRUN*, respectively. The posterior probability of the best lead SNP at these loci was relatively low with the best posterior probabilities of 0.23 for rs17361324 (*ADCY5*), and 0.11 for rs73420913 (*ILRUN*), respectively. Interestingly, however, the best candidate for causality at *PAX3* and *ADCY5* loci were the lead SNPs from the HISLA meta-analysis; for *ILRUN*, the FINEMAP and HISLA SNPs were in high LD (rs73420913 had an r^2^ = 0.96 with the lead HISLA SNP rs148899910), providing greater support for the prioritization of these SNPs for functional interrogation. For the *B4GALNT3* height locus, FINEMAP revealed six causal configurations. Four of the variants (rs11063185, rs215230, rs7303572, and rs11063184) with each configuration had a posterior probability >0.99 and contained only the variant itself in the 95th percentile credible set. One variant (rs215223) had a posterior probability of 0.93 and thus included two variants in the 95th percentile credible set. The sixth 95th percentile credible set had a lead variant with a posterior probability of 45% but contained a total of 1,621 additional variants, all of which had very small posterior probabilities (i.e., ≤0.05).

### Transferability of adult loci/signals from Hispanic/Latinos to consortia of other ancestral backgrounds

To assess how well the effect estimates are transferable to other populations, we looked up the BMI and height findings from Hispanic/Latinos in the AAAGC and GIANT meta-analysis results (Table 1). The BMI signal at the *ADCY5* locus (rs17361324) transferred to both AAAGC and GIANT with directional consistency (beta = 0.13–0.23) and at nominal significance (p < 5 × 10^−2^). The lead SNP (rs148899910) representing the BMI signal near *ILRUN* was not available in GIANT; the signal only appeared to be transferable to GIANT (at proxy SNP rs1573905, r^2^ = 0.96–1 in 1000 Genomes AMR and EUR; Table 1). The signal for height in *B4GALNT3* (rs215226) was directionally consistent and nominally significant in AAAGC only. In all cases, the effect sizes observed in GIANT and AAAGC were attenuated compared with the effect sizes from HISLA stage 1.

### Relevance of adult Hispanic/Latino anthropometric findings to childhood/adolescence

We looked up our novel HISLA findings in Hispanic/Latino children/adolescents using BMI-for-age and height-for-age *Z* scores, as well as a case-control study of childhood obesity. Two of the three novel BMI signals were directionally consistent with the anticipated effect on the odds of obesity during childhood/adolescence, one of which was nominally significant (rs17361324 at *ADCY5*; p = 2.2 × 10^−2^). None of the HISLA findings generalized at nominal significance with the BMI/height-for-age *Z* scores, but were directionally consistent with the corresponding effect in adulthood (Table S10). This may have been due to the small available sample size of Hispanic/Latino children/adolescents.

### Transferability of established anthropometric loci to Hispanic/Latino adults

We assessed how many established anthropometric loci, described previously in predominantly non-Hispanic/Latino European samples, could be transferred to Hispanic/Latino adults, in light of the available Hispanic/Latino sample size from stage 1. As shown in Table S11, the index SNPs at 336 of 1,247 (26.9%) previously reported BMI loci were suggestively transferable at nominal significance to Hispanic/Latinos. Of these BMI loci, 36 SNPs in the HISLA stage 1 displayed directional consistency with the literature and Bonferroni significance (Table S11). Furthermore, one BMI locus was genome-wide significant at the same published variant and another 12 BMI loci were genome-wide significant at another SNP within 1 Mb and in moderate to high LD (r^2^ ≥ 0.52 in AMR) with the reported index SNP (Table S7).

Table S12 shows that a slightly higher percentage of known height loci (1,177 of 3,806, or 30.9%) were transferable to Hispanic/Latinos. Of these loci, 124 SNPs were directionally consistent and Bonferroni significant (Table S12). Ten height loci were genome-wide significant at the same lead variant, and another 39 height loci were associated at genome-wide significance at another SNP within 1 Mb (0.05 ≤ r^2^ ≤ 0.98 in AMR; Table S7).

Finally, Tables S13–S15 show that 143 of 694 (20.6%) known WHRadjBMI in both sexes combined, 133 of 567 (23.5%) in women-only, and 28 of 173 (16.2%) in men-only loci were transferable to Hispanic/Latinos at nominal significance. Of these, a total of 15 loci were associated with WHRadjBMI at Bonferroni significance in the combined, women- or men-only analyses (Tables S13–S15). None of the index SNPs from the previous literature for WHRadjBMI reached genome-wide significance; however, we did observe genome-wide significant evidence for association of an SNP with WHRadjBMI in strong LD with the index variant (r^2^ = 0.92 in AMR) for the *HOXC13* signal (Table S7).

### Replication of five novel loci and 33 new signals in established loci for adult anthropometric traits from a trans-ancestral meta-analysis

Our secondary goal was to assemble a trans-ancestral meta-analysis of HISLA stage 1, AAAGC and GIANT consortia results to identify additional novel loci and fine-map established loci by leveraging differences in allele frequencies across populations (Figure 1). As anticipated, this trans-ancestral meta-analysis of HISLA, AAAGC, and GIANT revealed new insights, including 8 novel loci and 35 new signals in established loci that were associated at genome-wide significance (Table S16; Figures S12–S53) and independent of established SNPs within a 10 Mb region (Table 2). Of this set, 5 loci (3 BMI, 1 height, and 1 WHRadjBMI) and 33 signals in established loci (3 BMI, 28 height, and 2 WHRadjBMI) were generalized using the adult British subsample of the UKBB. In some cases, the significance in the trans-ancestral results were driven more by the AAAGC and/or HISLA consortia, which could explain the lack of association in the UKBB British subsample (Table S16; Figure S54).

**Table 2:** Novel loci and new signals in established loci by trait from a trans-ancestral meta-analysis of adult samples from the HISLA, AAAGC, and GIANT consortia

Trait	SNP rsid	Chr	Position (hg19)	Locus name^c^	Effect/other alleles	EAF			N	Unconditioned meta-analysis results				Conditioned on all known SNPs within 10 Mb region			UKBB (validation results)				
						HISLA	AAAGC	GIANT		Beta	SE	p value	HetISq	Beta	SE	p value	EAF	N	Beta	SE	p value
**Novel loci**^a^																					

BMI	rs4675117	2	227769794	*RHBDD1*	T/C	0.421	0.104	0.383	343,628	0.017	0.003	8.56 × 10^−8^	0	0.019	0.003	2.23 × 10^−9^	0.383	336,107	0.006	0.002	1.82 × 10^−2^
BMI	rs9860730	3	64701146	*ADAMTS9-AS2*	A/G	0.354	0.222	0.767	428,763	−0.016	0.003	1.67 × 10^−8^	0	−0.015	0.003	3.80 × 10^−8^	0.712	336,107	−0.008	0.003	4.54 × 10^−3^
BMI	rs150992	5	98275197	*CHD1-DT*	A/G	0.778	0.645	0.700	439,077	0.018	0.003	5.40 × 10^−10^	0	0.017	0.003	1.02 × 10^−8^	0.693	336,107	0.005	0.003	3.74 × 10^−2^
Height	rs17375290	1	61334177	*NFIA*	A/G	0.830	0.645	0.793	364,636	0.017	0.003	3.47 × 10^−8^	0	0.017	0.003	3.00 × 10^−8^	0.794	336,474	0.002	0.002	4.58 × 10^−1^
Height	rs10737541	1	168214098	*ANKRD36BP1*	T/G	0.399	0.645	0.196	319,809	−0.018	0.003	1.60 × 10^−9^	0	−0.018	0.003	3.16 × 10^−10^	0.226	336,474	−0.004	0.002	4.37 × 10^−2^
Height	rs4618485	6	73555917	*KCNQ5*	A/G	0.750	0.645	0.592	348,626	0.014	0.003	4.72 × 10^−8^	39.2	0.018	0.003	4.34 × 10^−12^	0.604	336,474	0.003	0.002	7.02 × 10^−2^
Height	rs17493997	8	82044302	*PAG1*	C/G	0.493	0.645	0.340	325,906	−0.015	0.003	3.42 × 10^−8^	0	−0.017	0.003	2.73 × 10^−10^	0.299	336,474	−0.0003	0.002	8.56 × 10^−1^
WHRadjBMI (sex combined)	rs16873543	6	45577134	*RUNX2*	T/C	0.716	0.645	0.767	209,552	−0.018	0.004	3.20 × 10^−6^	0	−0.022	0.004	9.65 × 10^−9^	0.724	484,563	−0.008	0.002	5.50 × 10^−4^

**New signals in established loci**^b^																					

BMI	rs10540	11	494662	*RNH1*	A/G	0.200	0.031	0.092	470,714	−0.021	0.004	1.01 × 10^−7^	0	−0.023	0.004	5.75 × 10^−9^	0.135	336,107	−0.007	0.004	4.16 × 10^−2^
BMI	rs4807179	19	1956035	*CSNK1G2*	A/G	0.494	0.218	0.525	309,507	0.020	0.003	2.75 × 10^−10^	0	0.018	0.003	1.52 × 10^−8^	0.632	336,107	0.014	0.002	1.06 × 10^−8^
BMI	rs4813428	20	21451848	*NKX2-2*	T/C	0.181	0.093	0.108	321,797	0.029	0.005	2.89 × 10^−10^	0	0.029	0.005	1.46 × 10^−10^	0.093	336,107	0.013	0.004	2.47 × 10^−3^
Height	rs4912122	1	19876438	*NKX2-2*	A/G	0.383	0.837	0.383	334,951	−0.015	0.003	6.33 × 10^−9^	0	−0.019	0.003	1.45 × 10^−13^	0.350	336,474	−0.012	0.002	1.58 × 10^−11^
Height	rs4425978	1	42243878	*HIVEP3*	T/C	0.389	0.189	0.580	351,587	0.014	0.003	2.14 × 10^−8^	0	0.016	0.003	3.63 × 10^−10^	0.533	336,474	0.008	0.002	5.41 × 10^−6^
Height	rs618555	1	86481084	*COL24A1*	T/C	0.241	0.064	0.353	320,239	0.019	0.003	5.47 × 10^−12^	0	0.016	0.003	2.90 × 10^−8^	0.311	336,474	0.008	0.002	1.67 × 10^−5^
Height	rs6545538	2	56217900	*MIR216A*	A/G	0.258	0.628	0.258	305,704	0.022	0.003	1.23 × 10^−13^	0	0.019	0.003	2.59 × 10^−11^	0.266	336,474	0.011	0.002	1.25 × 10^−8^
Height	rs2741311	2	233239743	*ALPP*	T/C	0.076	0.018	0.050	463,609	0.046	0.005	1.41 × 10^−21^	49.6	0.030	0.005	4.78 × 10^−10^	0.080	336,474	0.033	0.003	2.39 × 10^−24^
Height	rs6935954	6	26255451	*HIST1H2BH*	A/G	0.306	0.103	0.408	345,378	0.042	0.003	3.06 × 10^−59^	46.5	0.018	0.003	3.29 × 10^−12^	0.425	336,474	−0.027	0.002	1.10 × 10^−54^
Height	rs6900530	6	35280971	*DEF6*	T/C	0.094	0.385	0.042	123,137	−0.057	0.005	3.09 × 10^−28^	79.0	−0.036	0.005	6.75 × 10^−12^	0.027	336,474	−0.073	0.005	1.03 × 10^−42^
Height	rs9472006	6	43067487	*PTK7*	A/G	0.149	0.249	0.059	212,931	−0.027	0.005	4.37 × 10^−9^	0	−0.034	0.005	7.04 × 10^−14^	0.041	336,474	−0.013	0.004	2.86 × 10^−3^
Height	rs3822957	6	76607280	*MY O 6*	A/G	0.173	0.538	0.158	279,818	−0.023	0.003	4.88 × 10^−12^	84.4	−0.023	0.003	3.44 × 10^−12^	0.142	336,474	−0.015	0.002	1.98 × 10^−9^
Height	rs1342330	6	144065685	*PHACTR2*	A/T	0.601	0.738	0.508	353,259	0.014	0.003	1.70 × 10^−8^	0	0.017	0.003	6.95 × 10^−12^	0.520	336,474	0.006	0.002	8.84 × 10^−4^
Height	rs6936615	6	154355100	*OPRM1*	A/G	0.887	0.901	0.850	415,248	−0.018	0.003	2.47 × 10^−8^	0	−0.020	0.003	2.33 × 10^−9^	0.830	336,474	−0.003	0.002	1.73 × 10^−1^
Height	rs991946	6	166329862	*RP11-252P19.3*^c^	T/C	0.481	0.486	0.500	379,912	−0.019	0.002	1.28 × 10^−14^	36.7	−0.019	0.002	9.96 × 10^−15^	0.479	336,474	−0.013	0.002	3.92 × 10^−13^
Height	rs7816300	8	109787856	*TMEM74*	T/C	0.262	0.116	0.260	397,735	−0.015	0.003	2.24 × 10^−8^	0	−0.016	0.003	5.47 × 10^−9^	0.299	336,474	−0.002	0.002	3.94 × 10^−1^
Height	rs4520250	9	88924057	*TUT7*	A/C	0.235	0.069	0.433	295,945	0.015	0.003	3.80 × 10^−8^	55.9	0.016	0.003	1.48 × 10^−8^	0.339	336,474	0.010	0.002	4.36 × 10^−8^
Height	rs7029157	9	97000863	*snoU13*^c^	T/C	0.084	0.325	0.067	262,808	0.028	0.004	5.85 × 10^−11^	16.8	0.026	0.004	1.00 × 10^−9^	0.088	336,474	0.030	0.003	4.55 × 10^−22^
Height	rs12347744	9	97575273	*AOPEP*	T/C	0.102	0.015	0.033	454,111	−0.032	0.005	6.55 × 10^−11^	65.8	−0.030	0.005	6.06 × 10^−10^	0.061	336,474	−0.031	0.004	1.04 × 10^−17^
Height	rs7024254	9	109498129	*ZNF462*	A/G	0.255	0.721	0.207	324,351	0.017	0.003	1.04 × 10^−8^	23.7	0.036	0.003	6.10 × 10^−35^	0.204	336,474	0.010	0.002	6.50 × 10^−6^
Height	rs10119624	9	118305438	*DELEC1*	A/G	0.612	0.437	0.683	353,374	0.021	0.003	2.56 × 10^−16^	31.1	0.022	0.003	8.02 × 10^−17^	0.671	336,474	0.012	0.002	3.20 × 10^−10^
Height	rs2902635	10	105476045	*SH3PXD2A*	T/G	0.710	0.382	0.821	308,782	−0.021	0.003	1.59 × 10^−12^	0	−0.017	0.003	1.62 × 10^−8^	0.805	336,474	−0.015	0.002	7.65 × 10^−12^
Height	rs17659078	11	2284590	*ASCL2*	A/C	0.211	0.149	0.258	354,931	0.019	0.003	1.78 × 10^−10^	9.0	0.016	0.003	3.45 × 10^−8^	0.273	336,474	0.004	0.002	2.24 × 10^−2^
Height	rs11605693	11	122837037	*JHY*	T/C	0.591	0.384	0.448	380,447	−0.017	0.002	3.44 × 10^−12^	0	−0.018	0.002	3.07 × 10^−14^	0.447	336,474	−0.013	0.002	4.45 × 10^−13^
Height	rs621794	11	125849462	*CDON*	A/G	0.500	0.430	0.475	380,049	−0.014	0.002	1.46 × 10^−8^	0	−0.014	0.002	7.29 × 10^−9^	0.429	336,474	−0.009	0.002	1.76 × 10^−7^
Height	rs11221442	11	128577624	*FLI1*	C/G	0.171	0.109	0.217	352,360	−0.022	0.003	2.03 × 10^−12^	78.8	−0.023	0.003	2.03 × 10^−13^	0.252	336,474	−0.008	0.002	2.59 × 10^−5^
Height	rs12300112	12	103147575	*LINC00485*	C/G	0.075	0.242	0.008	154,014	0.041	0.006	4.31 × 10^−13^	45.2	0.038	0.006	3.11 × 10^−11^	0.027	336,474	0.038	0.005	4.61 × 10^−12^
Height	rs11616067	12	116393174	*MED13L*	A/G	0.841	0.904	0.750	327,941	0.021	0.003	6.38 × 10^−12^	21.0	0.018	0.003	2.80 × 10^−9^	0.768	336,474	0.012	0.002	6.01 × 10^−9^
Height	rs17197170	14	21977962	*METTL3*	A/G	0.931	0.960	0.842	310,344	−0.026	0.004	5.40 × 10^−12^	64.9	−0.025	0.004	3.74 × 10^−11^	0.828	336,474	−0.018	0.002	3.07 × 10^−14^
Height	rs11076551	16	51109492	*RP11-883G14.4*^c^	A/G	0.341	0.343	0.267	434,688	0.014	0.003	2.14 × 10^−8^	21.6	0.015	0.003	5.87 × 10^−9^	0.376	336,474	0.009	0.002	2.51 × 10^−7^
Height	rs12918773	16	89741403	*SPATA33*	A/G	0.121	0.036	0.149	279,104	−0.024	0.004	7.45 × 10^−9^	46.1	−0.024	0.004	3.50 × 10^−9^	0.112	336,474	−0.024	0.003	4.71 × 10^−19^
Height	rs1346490	19	7244233	*INSR*	A/C	0.546	0.611	0.500	333,896	0.015	0.003	3.99 × 10^−9^	12.3	0.014	0.003	4.86 × 10^−8^	0.620	336,474	0.009	0.002	1.67 × 10^−7^
Height	rs17457472	19	17493610	*PLVAP*	A/C	0.037	0.062	0.028	356,712	−0.051	0.007	5.70 × 10^−14^	0	−0.042	0.007	9.89 × 10^−10^	0.040	336,474	−0.019	0.004	1.12 × 10^−5^
WHRadjBMI (sex combined)	rs17099388	5	142095250	*FGF1*	A/G	0.166	0.256	0.008	105,460	0.039	0.007	3.14 × 10^−9^	0	0.037	0.007	2.47 × 10^−8^	0.041	484,563	0.025	0.005	2.00 × 10^−7^
WHRadjBMI (sex combined)	rs7975017	12	26428793	*SSPN*	T/C	0.303	0.717	0.167	267,044	−0.021	0.004	7.57 × 10^−9^	5.5	−0.021	0.004	1.05 × 10^−8^	0.239	484,563	−0.014	0.002	2.00 × 10^−9^

Chr, chromosome; EAF, effect allele frequency; HetIsq, heterogeneity I^2^; N, sample size; WHRadjBMI, waist-to-hip ratio adjusted for BMI; AAAGC, African American Anthropometry Genetics Consortium; GIANT, Genetic Investigation of Anthropometric Traits Consortium.

a Each novel locus was defined by the absence of known (previously published) SNPs within 1 Mb (±500 kb) of the lead SNP.

b Each known locus was defined by a 1 Mb region around previously identified SNP(s) for the indicated trait; the known SNP(s), p < 5 × 10^−8^, at each established locus can be found in Table S16.

c Human Genome Organisation approved gene names, unless otherwise indicated.

We looked up the findings from our trans-ancestral meta-analyses in the sample of Hispanic/Latino children/adolescents (Table S17). We found that 2 of the 7 BMI and height trans-ancestral loci, and 17 of the 33 trans-ancestral BMI/height signals in established loci, were directionally consistent between their adult directions of association and the BMI/height-for-age *Z* scores in children/adolescents. However, this amount of directional consistency was not more than what would have been expected by chance alone (p_binomial_ > 0.10). Four trans-ancestral SNPs were associated at nominal significance in the child/adolescent sample, each having been already replicated in UKBB (Table S16). Three of these four loci were directionally consistent in the childhood/adolescence results with the trans-ancestral adult findings (Table S17).

### Fine-mapping of trans-ancestral anthropometric findings

We also fine-mapped our trans-ancestral findings (Table S21) using FINEMAP to pinpoint individual variants and genes within each locus region that have a direct effect on the trait. FINEMAP uses a shotgun stochastic search algorithm^84^ that iterates through causal configurations of SNPs by concentrating efforts on the configurations with non-negligible probability. Within a 1 Mb region, we report (1) the causal configuration of SNPs for a given trait that had the highest posterior probability and (2) the posterior probability of being causal for each of the SNPs.

For four of the five trans-ancestral loci (three BMI loci and one WHRadjBMI locus), there was one SNP within the configuration with the highest posterior probability. For the height locus near *ANKRD36BP1*, there were two SNPs in the configuration with the highest posterior probability. In all five loci, the SNP with the highest posterior probability from each of these credible sets was either the exact SNP with the strongest GWAS evidence or in high LD (r^2^ between 0.70 and 0.99 in each ancestry) with the lead GWAS SNP. Two of these five regions had strong prioritization given high posterior probabilities (≥0.8) and small 95th percentile credible sets: (1) for BMI, the *CHD1-DT* region had a posterior probability of 0.88 for rs150992 with three SNPs in the credible set, and (2) for height, the *ANKRD36BP1* region had a posterior probability of 0.93 for rs10737541 with five SNPs in the credible set. From the functional annotations (Tables S22 and S23), we find that all three of the BMI loci, the height loci, and WHRadjBMI loci have enhancer marks and eQTLs, most of which are in highly relevant tissues, e.g., adipose, brain, muscle, thyroid.

For the other trans-ancestral loci, the posterior probabilities were lower, between 0.09 and 0.42, yet four loci (rs9860730, rs17375290, rs4324883, and rs9463108) still had relatively few SNPs (<10) in the 95th percentile credible sets, suggesting a narrow window (combination of variants) around the causal variant. For example, functional annotations of rs17375290, the lead GWAS SNP in the NFIA locus associated with height, show it to have promoter markers in muscle, CADD score of 13.29 (CADD >10 ranks variants among the top 10% potentially deleterious), and an eQTL with *FGGY* in osteoclast tissue (Tables S22 and S23). Three of the other SNPs in the credible set (rs599989, rs1762881, and rs17121184) have nominally significant (p = 0.01–0.005) eQTLs with *FGGY* in osteoclast tissue, but are not in high LD with rs17375290 (r^2^ = 0.03–0.1). Diseases associated with *FGGY* include autosomal recessive lateral sclerosis and spastic paraplegia type 7, which are known to affect height.

Within the 33 trans-ancestral signals in known loci, 31 had configurations with more than 1 putative causal SNP (e.g., more than 1 credible set). This made sense given these are loci with multiple independent signals, as described by our earlier conditional analyses. Among the putative causal SNPs within each locus, there were a number of SNPs that represented known signals (either the exact SNP or something in high LD among all ancestries). We found that, for many of these, the credible sets contained <10 SNPs. Among the 33 signals in known loci, 26 included a putative causal SNP that is the lead GWAS SNP reported here or an SNP in high LD (r^2^ > 0.75) with the lead GWAS SNP, suggesting causality for this signal in general, although perhaps maybe not initially described at the most-putatively causal SNP(s). For these putatively causal SNPs, the posterior probabilities ranged from 0.09 to 1. Twenty-two of these SNPs had 95th percentile credible sets that contained <10 SNPs and 15 also had posterior probability ≥0.8.

Many have functional annotations that support the fine-mapping results (Tables S22 and S23). For example, we find eQTLs for the three BMI signals and enhancer marks for rs4807179 in relevant tissues, including adipose, brain, muscle, and/or thyroid. The lead SNPs of these credible sets had posterior probabilities >0.75 and the credible sets included <10 SNPs. Of the 28 identified height signals, we find 13 putatively causal SNPs that are the lead GWAS SNP, or are in high LD (r^2^ > 0.75) with it, have <10 SNPs in the credible set and have eQTLs in relevant tissues, including muscle, thyroid, adipose, lung, and osteoclasts. Some also have promoter or enhancer marks in some of the same tissues. For the two WHRadjBMI signals, both have three SNPs in the most probable causal configurations. One of these causal SNPs for each region is either the lead GWAS SNP (rs7975017) or an SNP in high LD (rs17099388 and rs6895040 LD: AFR r^2^ = 1.0; AMR r^2^ = 1.0; EUR r^2^ = 1.0), has a posterior probability ≥0.95, and is the only SNP in the credible set. Furthermore, for rs7975017, we find eQTLs in thyroid for multiple genes (*BHLHE41*, *SSPN*, and AC022509.3 from *GTEx*) and enhancer marks in multiple tissues including those related to the WHRadjBMI trait, e.g., thyroid, muscle, fat, bone, and adrenal gland. Overall, across many of the loci and secondary signals, FINEMAP revealed SNPs with somewhat strong prioritization (posterior probability ≥0.8) and, at some loci, putatively causal SNPs in small 95th percentile credible sets, thus demonstrating the utility of trans-ancestral approaches to fine-mapping GWAS loci.

### Trans-ancestral findings to account for population structure in previous GWAS

**Figure 3:**
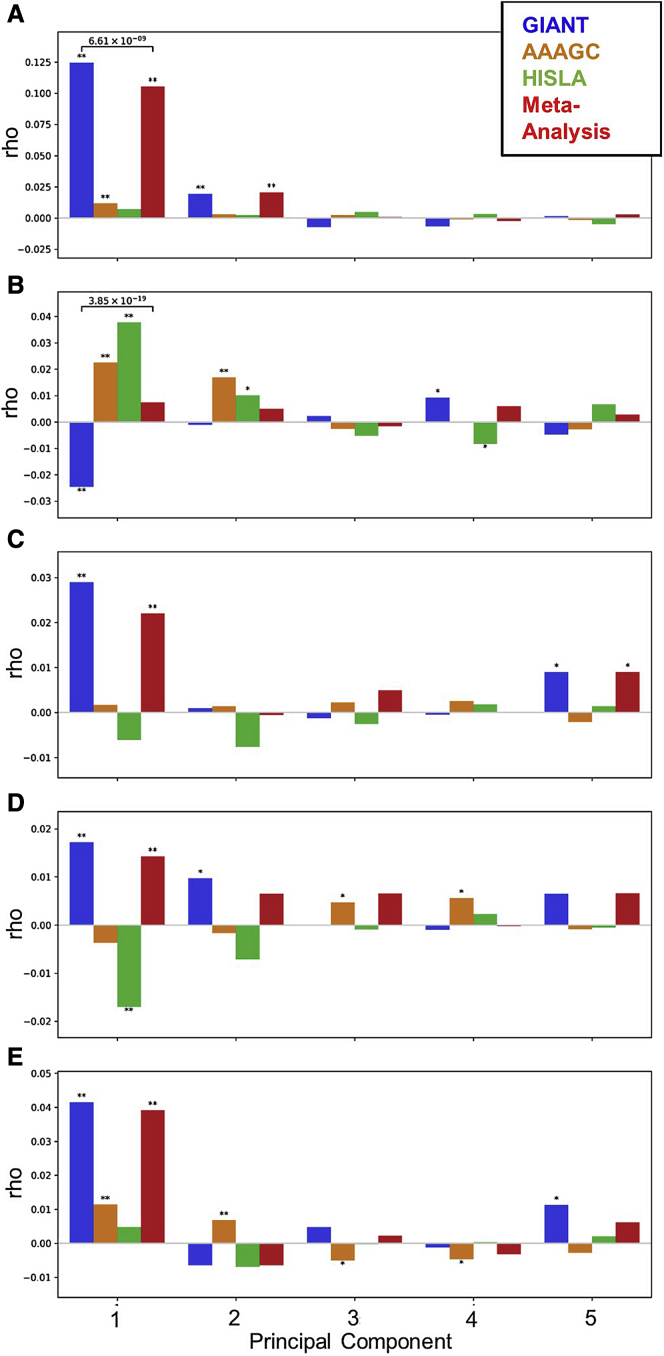
Correlations (rho) between effect estimates and the loadings of principal components 1–5 in each consortium and the meta-analysis of all three consortia by trait (A) Height, (B) BMI, (C) WHRadjBMI for men and women combined, (D) WHRadjBMI for women only, and (E) WHRadjBMI for men only. HISLA, Hispanic/Latino Anthropometry Consortium; AAAGC, African American Anthropometry Genetics Consortium; Genetic Investigation of Anthropometric Traits; WHRadjBMI, waist-to-hip ratio adjusted for BMI.

Previous height GWAS utilizing only European ancestry samples are known to exhibit signatures of residual stratification, which manifest in effect size estimates of height-associated SNPs being correlated with geographical structure in Europe.^85^, ^86^, ^87^, ^88^ In theory, this bias should be lessened with addition of non-European samples in a trans-ancestry GWAS, since geographical structure across different continental ancestries are not expected to be correlated with each other. We demonstrate this hypothesis empirically using the HISLA data. The first two PCs in the PCA of European populations (Figure S55) reflect geographical or population structure in Europe, corresponding to the north-south and southeast-southwest axes of variation, respectively. We found that the bias in effect size estimates due to stratification is most obvious for height as this phenotype is known to differ across Europe.^85^^,^^89^^,^^90^ Effect sizes on height estimated from the GIANT and our trans-ancestral meta-analysis were both highly correlated with the loadings of the first PCA (rho = 0.125, p = 3.2 × 10^−94^ in GIANT; rho = 0.105, p = 3.4 × 10^−70^ in meta-analysis). The correlation was much lower in AAAGC and HISLA (rho = 0.012, p = 2.17 × 10^−4^ in AAAGC; rho = 0.007, p = 9.2 × 10^−2^ in HISLA; Figure 3A). Importantly, the magnitude of correlation was lessened in meta-analysis compared GIANT alone (p = 6.6 × 10^−9^), consistent with our hypothesis. Other traits were not *a priori* known to be as differentiated across Europe as height, and thus the degree of correlation between effect sizes and PC loadings are much lower in GIANT (e.g., rho = −0.025 for BMI; Figures 3B–3E).

## Discussion

Hispanic/Latinos are a unique population with continental admixture from the Americas, Africa, and Europe,^10^, ^11^, ^12^, ^13^, ^14^ and yet are underrepresented in GWAS. Herein, we present results from a large-scale meta-analysis of anthropometric traits on an ancestrally diverse sample of Hispanic/Latino adults (Figures S1–S3). We have assembled a landmark consortium of Hispanics/Latinos to discover and map a total of 6 novel loci and 36 novel signals using both Hispanic/Latino population-specific and trans-ancestral discovery efforts (Figure 1). Numerous previously-reported anthropometric-SNP associations were suggestively (at nominal significance) or strongly (at Bonferroni significance) transferable to Hispanic/Latino adults. For example, between 16% and 31% of anthropometric variants transferred to Hispanic/Latino adults, depending on the given trait or sex-specific analyses conducted (Tables S11–S13). In total, 67 previously reported loci reached genome-wide significance in our Hispanic/Latino adult sample at the same index or another lead SNP, the majority of which were in high LD in 1000 Genomes EUR or AMR (Table S7). Moreover, we observed that four of seven of our HISLA findings were transferable to other ancestral populations at nominal significance.

We note that, even though these findings provide additional evidence for transferability of common loci for anthropometrics,^91^ still a number of previously reported anthropometric loci may not be transferable to this population in part due to variability in allele frequencies, effect sizes across ancestral populations, or our relatively smaller sample compared with European consortia.^55^ Thus, absence of generalization does not equate to a lack of relevance to Hispanic/Latino adults or children, especially given that Hispanic/Latinos are under-studied population in genetic research and larger/comparable sample sizes are currently unavailable.

Our conditional and fine-mapping analyses revealed 36 signals in established anthropometric loci, which independently replicated in HISLA stage 2 or the UKBB British subsample. In addition, our lead SNPs for the BMI signals discovered at *ADCY5* (from the HISLA meta-analysis) and *ADAMTS9-AS2* (from the trans-ancestral meta-analysis) are both nominally associated with childhood obesity status aged between 2 and 18 years. Three of our trans-ancestral signals in established height loci also displayed association with height-for-age *Z* scores in children/adolescents aged between 5 and 18 years. These observations support the premise that diverse and trans-ancestral studies represent a valuable tool for leveraging ancestral differences and similarities both within and across populations to identify multiple signals in established association regions, identify putative variants that may account for some of the missing heritability of complex diseases, or reveal promising genes and SNPs for functional follow-up.

**Figure 4:**
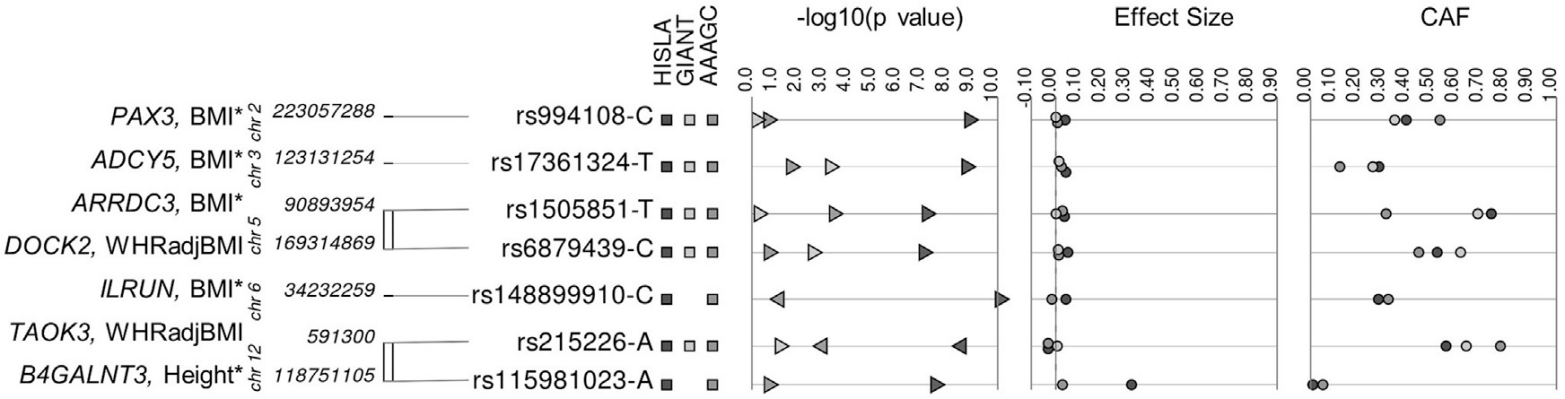
Variability in HISLA stage 1 + 2, AAAGC, and GIANT p values, effect sizes, and coded allele frequencies for genome-wide significant anthropometric loci from HISLA stage 1 AAAGC, African American Anthropometry Genetics Consortium; BMI, body mass index; CAF, coded allele frequency; GIANT, Genetic Investigation of Anthropometric Traits; HISLA, Hispanic/Latino Anthropometry Consortium; WHRadjBMI, waist-to-hip ratio adjusted for BMI. ∗SNPs that remained significant (p < 5 × 10^−8^) in HISLA stage 1 + 2.

In light of the notable ancestral, geographical or environmental diversity of the samples analyzed in our meta-analyses, we observed evidence of allele frequency differences for many of our Hispanic/Latino (Figure 4) and trans-ancestry findings (Figure S53). Similar to reports from other diverse genome-wide analyses,^55^ this allele frequency heterogeneity may explain heterogeneity in effects seen across consortia in our trans-ancestral HISLA, AAAGC, and GIANT meta-analysis (e.g., *IGF2BP2* I^2^ = 78.7; *MYO6* with I^2^ = 84.4, Tables 2 and S16). Our use of fixed-effect meta-analyses may have failed to identify loci with effect heterogeneity unrelated to allele frequency or LD differences across populations; future studies should address this limitation by considering trans-ancestral random-effects meta-analysis, local ancestry and haplotype analyses as these studies explore sources of heterogeneity in large, diverse datasets. These observations reinforce how studies of one predominant ancestry group, such as Europeans, may fail to identify additional loci or, more likely, new signals in known loci that have allele frequency differences across ancestral populations.

Residual uncorrected stratification in GWAS could result in biased estimates of effect sizes.^34^ For example, effect sizes on height from GIANT were reported to be significantly correlated with north-south axis of variation in Europe, suggesting residual uncorrected stratification,^85^, ^86^, ^87^ which we also observe here. Note that the residual stratification is subtle, and while the effect sizes may be biased, this does not imply that the identified associations are spurious. For example, compared with effect sizes on height from UKBB, which is based on a single homogeneous population and results in better control of population stratification, the genetic correlation between GIANT and UKBB was 0.94.^85^

Of the three traits studied here, height is the most stratified in Europe. The correlation coefficient between effect sizes on height and PC loadings reached 0.125 in the GIANT only for PC1, while it was much smaller for other traits (e.g., the maximum |*rho*| = 0.042 in GIANT on WHR using only males on PC1). The decrease in bias in the trans-ancestral meta-analysis was also obvious in height. The correlation with PC1 was non-significant in HISLA (*rho* = 0.007) and statistically significant but weak in AAAGC (*rho =* 0.012), consistent with a decreased impact of European population stratification on the estimate of effect size in AAAGC and HISLA. This decreased correlation could be due to large non-European ancestries in these populations (African and Native American, respectively) that make these populations affected by population stratification in Europe; it could also be that, by using European ancestry-based loadings, we are less likely to detect non-European based population stratification patterns or that smaller sample sizes in these cohorts result in greater noise in effect size estimates. Regardless of the reason, compared with GIANT alone, trans-ancestral meta-analysis of the three cohorts showed less impact of uncorrected stratification on effect size estimates, even though the sample sizes in AAAGC and HISLA are comparably small. For other traits, the conclusions are qualitatively similar: that trans-ancestral meta-analysis lessened the bias due to stratification, even though the bias in GIANT was not as strong in the first place.

Gene expression and bioinformatic analyses of our population-specific (Tables S18–S20) and trans-ancestral findings in newly discovered loci (Tables S22 and S23) revealed important insights into the underlying biology of obesity, bone development, and growth. For example, the previously reported BMI locus *ILRUN* has also been associated with adult height^92^^,^^93^ and height change during puberty.^94^ The previously described BMI signal was lead at rs205262, an eQTL for another gene within the region (*SNRPC*) in European ancestry samples.^33^ A second signal (rs75398113) has also been reported at *SNRPC* for extremes of the BMI distribution.^95^ Yet, our signal led by rs148899910 is more than 300 kb away and in low LD with these two index SNPs (r^2^ = 0.01–0.05 in 1000 Genomes AMR). More recently, rs148899910 has been associated with height in Korean women.^96^ Furthermore, variants in high LD with rs148899910 in 1000 Genomes AMR are associated with type 2 diabetes in individuals of East Asian ancestry^97^ (rs4711389 has r^2^ = 0.9 in 1000 Genomes AMR with rs148899910), and with BMI-adjusted waist circumference in individuals of European ancestry (rs202228093 and rs2780226 each have an r^2^ > 0.7 with rs148899910 in 1000 Genomes AMR).^44^^,^^98^ Using whole blood gene expression data from 606 participants of the Cameron County Hispanic Cohort, we find evidence that our BMI signal at rs148899910 is an eQTL for increased gene expression of *C6orf1* (p = 3 × 10^−7^) and not any other genes in the region (Table S18). Taken together, this signal shows associations across a wide array of anthropometric phenotypes.

In general, the lead SNPs from our HISLA-only meta-analyses appear relatively benign (not pathogenic) based on CADD and FATHMM-XF scores (Table S20). Yet, all SNPs potentially change motifs. Both rs17361324 (*ADCY5*) and rs215226 (*B4GALNT3*) have enhancer and promoter histone marks and eQTLs in the respective genes in relevant tissues. For BMI, there is an eQTL for rs17361324-ADCY5 in thyroid, and *ADCY5* has been previously associated with type 2 diabetes,^99^ BMI,^100^ central obesity traits,^39^ height,^47^ birth outcomes,^101^, ^102^, ^103^ and a number of other phenotypes. In addition, rs17361324 is proximate to an *ADCY5* intronic variant (rs1093467, r^2^ = 0.3 in 1000 Genomes AMR) that is highly conserved across species (Haploreg v.4.1). For height, there is an eQTL for rs215226-B4GALNT3 in aortic (coronary) and tibial nerves. The lead SNP for the height signal in *B4GALNT3*, rs215226, has enhancer histone marks in bone and muscle, and promoter marks in muscle tissue. In addition, the variant rs215226 (*B4GALNT3*) has a posterior probability of 1 in FINEMAP analyses (see Table S9). Other interesting information about these regions is provided in Table S19.

The lead SNPs in our trans-ancestral loci were mainly located in intronic and intergenic regions (Table S22) and were benign. One exception was the locus *C11orf63* associated with height led by rs11605693, which showed pathogenic scores for CADD and FATHMM-XF (CADD score = 17.1 and FATHMM-XF score = 0.87). This lead SNP has an eQTL in *C11orf63* for adipose, tibial nerve, and testis. *C11orf63*, junctional cadherin complex regulator, is responsible for ependymal cells that line the brain and spinal cord.

Among the trans-ancestral findings, a BMI signal in the established locus *RNH1* was led by rs10540 (posterior probability of 0.82), and is an eQTL for a wide range of tissues and genes (see Tables S21 and S23). Another signal in a known locus for height, led by rs12918773, has a posterior probability of 0.98 and is one of four casual variants suggested from fine-mapping in the locus (Table S21), has an eQTL (in lung, thyroid, tibial nerve and artery, breast, testis) with *CDK10*, a gene also associated with growth retardation.^104^ In addition, rs1342330 led the newly discovered signal in a known height locus, and has a low regulomeDB score at 2b, and several enhancer and promoter histone marks in relevant tissues (Tables S22). As an intronic variant, it is an eQTL in the pancreas with *PHACTR2* (Table S23), a gene associated with body dysmorphic disorder.^105^ While many of our discovered loci/signals appeared to be benign based on CADD and FATHMM-XF scores, they still show enhancer and promoter histone marks in trait-relevant tissues, such as adipose tissue, bone, muscle, thymus, brain, and adrenal gland.

As described above, in this study we were able to (1) discover six additional loci with a notably smaller analytic size than other anthropometric consortia, such as GIANT. We also (2) discovered 36 signals in established loci in HISLA or our trans-ancestral meta-analysis, and (3) generated trans-ancestral effect estimates with better control for population structure. Taken together, these findings indicate the added value of building large, more diverse GWAS in the near future.

Large-scale analyses of diverse populations hold great potential for advancing the field of genetic epidemiology.^55^ This study illustrates how studying admixed populations, such as Hispanics/Latinos, and highlighting them in trans-ancestral epidemiologic investigations, can yield additional insights into the genetic architecture of anthropometric traits. Future discovery efforts in Hispanic/Latino populations and with other ancestrally diverse populations will help address the concerning research gap between who is studied and who is affected by conditions, such as obesity, to the benefit of both public health and precision medicine.

## Data Availability

The HISLA meta-analysis results (GWAS catalog access IDs: height, GCST90095033; BMI, GCST90095034; WHRadjBMI [sex combined], GCST90095035; WHRadjBMI [men], GCST90095036; WHRadjBMI [women], GCST90095037), and the trans-ancestral HISLA, AAAGC, and GIANT meta-analysis results (GWAS catalog access ID: height, GCST90095038; BMI, GCST90095039; WHRadjBMI [sex combined], GCST90095040; WHRadjBMI [men], GCST90095041; WHRadjBMI [women], GCST90095042) are available through the NHGRI-EBI catalog.

## Web resources

LocusZoom, https://locuszoom.org.

GWAS catalog, https://www.ebi.ac.uk/gwas/.

Eigensoft version 7.2.1, https://github.com/DReichLab/EIG/archive/v7.2.1.tar.gz.

NHGRI-EBI catalog, https://www.ebi.ac.uk/gwas/.

## Supplemental information

Document S3. Supplemental data

Document S4. Article plus supplemental information

## Footnotes

Supplemental information can be found online at https://doi.org/10.1016/j.xhgg.2022.100099.
