# Supplementary material for: Ancestral diversity improves discovery and fine-mapping of genetic loci for anthropometric traits—The Hispanic/Latino Anthropometry Consortium
Source: HGG Adv. 2022 Mar 11;3(2):100099. doi: 10.1016/j.xhgg.2022.100099 (PMC8990175; doi:10.1016/j.xhgg.2022.100099)
Supplement: Document S1. Figures S1–S55 [file mmc1.pdf]

HGGA, Volume *3*

Supplemental information

Ancestral diversity improves discovery and fine-mapping of genetic loci for anthropometric traits—The Hispanic/Latino Anthropometry Consortium

Lindsay Fernández-Rhodes, Mariaelisa Graff, Victoria L. Buchanan, Anne E. Justice, Heather M. Highland, Xiuqing Guo, Wanying Zhu, Hung-Hsin Chen, Kristin L. Young, Kaustubh Adhikari, Nicholette D. Palmer, Jennifer E. Below, Jonathan Bradfield, Alexandre C. Pereira, LáShauntá Glover, Daeeun Kim, Adam G. Lilly, Poojan Shrestha, Alvin G. Thomas, Xinruo Zhang, Minhui Chen, Charleston W.K. Chiang, Sara Pulit, Andrea Horimoto, Jose E. Krieger, Marta Guindo-Martínez, Michael Preuss, Claudia Schumann, Roelof A.J. Smit, Gabriela Torres-Mejía, Victor Acuña-Alonzo, Gabriel Bedoya, Maria-Cátira Bortolini, Samuel Canizales-Quinteros, Carla Gallo, Rolando González-José, Giovanni Poletti, Francisco Rothhammer, Hakon Hakonarson, Robert Igo, Sharon G. Adler, Sudha K. Iyengar, Susanne B. Nicholas, Stephanie M. Gogarten, Carmen R. Isasi, George Papnicolaou, Adrienne M. Stilp, Qibin Qi, Minjung Kho, Jennifer A. Smith, Carl D. Langefeld, Lynne Wagenknecht, Roberta Mckean-Cowdin, Xiaoyi Raymond Gao, Darryl Nousome, David V. Conti, Ye Feng, Matthew A. Allison, Zorayr Arzumanyan, Thomas A. Buchanan, Yii-Der Ida Chen, Pauline M. Genter, Mark O. Goodarzi, Yang Hai, Willa Hsueh, Eli Ipp, Fouad R. Kandeel, Kelvin Lam, Xiaohui Li, Jerry L. Nadler, Leslie J. Raffel, Kathryn Roll, Kevin Sandow, Jingyi Tan, Kent D. Taylor, Anny H. Xiang, Jie Yao, Astride Audirac-Chalifour, Jose de Jesus Peralta Romero, Fernando Hartwig, Bernando Horta, John Blangero, Joanne E. Curran, Ravindranath Duggirala, Donna E. Lehman, Sobha Puppala, Laura Fejerman, Esther M. John, Carlos Aguilar-Salinas, Noël P. Burtt, Jose C. Florez, Humberto García-Ortíz, Clicerio González-Villalpando, Josep Mercader, Lorena Orozco, Teresa Tusié-Luna, Estela Blanco, Sheila Gahagan, Nancy J. Cox, Craig Hanis, Nancy F. Butte, Shelley A. Cole, Anthony G. Comuzzie, V. Saroja Voruganti, Rebecca Rohde, Yujie Wang, Tamar Sofer, Elad Ziv, Struan F.A. Grant, Andres Ruiz-Linares, Jerome I. Rotter, Christopher A. Haiman, Esteban J. Parra, Miguel Cruz, Ruth J.F. Loos, and Kari E. North

Supplemental Materials

Baependi Heart Study

The Baependi Heart Study was supported through a collaborative effort by FAPESP and Brazil Health Ministry (PROADI). ACP was supported by NHLBI R01HL141881-01A1.

California Breast Cancer Studies

The Northern California Breast Cancer Family Registry (NC-BCFR) is supported by grant UM1 CA164920 from the NCI. The content of this manuscript does not necessarily reflect the views or policies of the National Cancer Institute or any of the collaborating centers in the Breast Cancer Family Registry (BCFR), nor does mention of trade names, commercial products, or organizations imply endorsement by the United States Government or the BCFR. The San Francisco Bay Area Breast Cancer Study (SFBCS) was supported by grants R01 CA63446 and R01 CA77305 from the National Cancer Institute, grant DAMD17-96-1-6071 from the U.S. Department of Defense, and grant 7PB-0068 from the California Breast Cancer Research Program.

Computation for part of this work was supported by the University of Southern California's Center for Advanced Research Computing (https://carc.usc.edu).

Consortium for the Analysis of the Diversity and Evolution of Latin America

The CANDELA consortium has received funding from: the National Natural Science Foundation of China (#31771393), the Scientific and Technology Committee of Shanghai Municipality (18490750300), Ministry of Science and Technology of China (2020YFE0201600), Shanghai Municipal Science and Technology Major Project (2017SHZDZX01) and the 111 Project (B13016), the Leverhulme Trust (F/07 134/DF), BBSRC (BB/I021213/1), the Excellence Initiative of Aix-Marseille University - A*MIDEX (a French "Investissements d'Avenir" programme), the National Natural Science Founddation of China

(#31771393), Universidad de Antioquia (CODI sostenibilidad de grupos 2013- 2014 and MASO 2013-2014), Conselho Nacional de Desenvolvimento Científico e Tecnológico, Fundação de Amparo à Pesquisa do Estado do Rio Grande do Sul, and Fundação de Aperfeiçoamento de Pessoal de Nível Superior (Apoio a Núcleos de Excelência Program).

Family Investigation of Nephropathy and Diabetes

The Family Investigation of Nephropathy and Diabetes study was supported by grants U01DK57292, U01DK57329, U01DK057300, U01DK057298, U01DK057249, U01DK57295, U01DK070657, U01DK057303, and U01DK57304 from NIDDK and, in part, by the Intramural Research Program of the NIDDK. Support was also received from the NHLBI grants U01HL065520, U01HL041654, and U01HL041652. This project has been funded in whole or in part with federal funds under contract N01-CO-12400 and the Intramural Research Program of the NIH, NCI, Center for Cancer Research. This work was also supported by the National Center for Research Resources for the General Clinical Research Center grants: Case Western Reserve University, M01-RR-000080; Wake Forest University, M01-RR-07122; Harbor-University of California, Los Angeles Medical Center, M01-RR-00425; College of Medicine, University of California, Irvine, M01-RR-00827–29; University of New Mexico, HSC M01-RR-00997; and Frederic C. Bartter, M01-RR-01346. Computing resources were provided, in part, by the Wake Forest School of Medicine Center for Public Health Genomics. The funders had no role in study design, data collection and analysis, decision to publish, or preparation of the manuscript.

Hispanic Community Health Study/Study of Latinos

The Hispanic Community Health Study/Study of Latinos was carried out as a collaborative study supported by contracts from the National Heart, Lung, and Blood Institute (NHLBI) to the University of North Carolina (N01-HC65233), University of Miami (N01-HC65234), Albert Einstein College of Medicine (N01-HC65235), Northwestern University (N01-

HC65236), and San Diego State University (N01-HC65237). The following Institutes/Centers/Offices contribute to the HCHS/SOL through a transfer of funds to the NHLBI: National Institute on Minority Health and Health Disparities, National Institute on Deafness and Other Communication Disorders, National Institute of Dental and Craniofacial Research, National Institute of Diabetes and Digestive and Kidney Diseases, National Institute of Neurological Disorders and Stroke, NIH Institution-Office of Dietary Supplements. The Genetic Analysis Center including several authors on this manuscript (i.e. SMS, CCL, AMS) at the University of Washington was supported by NHLBI and NIDCR contracts (HHSN268201300005C AM03 and MOD03).

The Lundquist Institute for Biomedical Innovation-Affiliated Studies

Support for the Multi-Ethnic Study of Atherosclerosis (MESA) projects are conducted and supported by the National Heart, Lung, and Blood Institute (NHLBI) in collaboration with MESA investigators. Support for MESA is provided by contracts 75N92020D00001, HHSN268201500003I, N01-HC-95159, 75N92020D00005, N01-HC-95160, 75N92020D00002, N01-HC-95161, 75N92020D00003, N01-HC-95162, 75N92020D00006, N01-HC-95163, 75N92020D00004, N01-HC-95164, 75N92020D00007, N01-HC-95165, N01-HC-95166, N01-HC-95167, N01-HC-95168, N01-HC-95169, UL1-TR-000040, UL1-TR-001079, and UL1-TR-001420, UL1TR001881, DK063491, and R01HL105756. The authors thank the other investigators, the staff, and the participants of the MESA study for their valuable contributions. A fill list of participating MESA investigators and institutes can be found at http://www.mesa-nhlbi.org.

This research was also supported by the Mexican-American Coronary Artery Disease (MACAD) contracts R01-HL088457, R01-HL-60030; Hypertension and Insulin Resistance (HTN-IR) contracts R01-HL067974, R01-HL-55005, R01-HL 067974, NIDDM-Athero contract HL-055798, Insulin Resistance Atherosclerosis Study (IRAS) contracts HL-047887, HL-047889,

HL-047890, HL-47902, and the Insulin Resistance Atherosclerosis Family Study (IRAS-FS) contracts HL-060944, HL-061019, and HL-060919. The provision of genotyping and data analysis was supported by the GUARDIAN Consortium contracts DK-085175 and DK-118062. Work related to insulin clearance in HTN-IR, MACAD, and NIDDM-Athero was supported by contract DK-079888. This work was also supported in part by the Genetics of Latinos Diabetic Retinopathy (GOLDR) Study grant EY14684. This study was supported in part by the National Institutes of Health, National Heart, Lung, and Blood Institute contract R01 HL0767711. Research support for the Translation cohorts was provided by U10-EY-011753 (LALES), R01-EY022651 (MAGGS, Mexican American Glaucoma Genetic Study), P30-EY001792 (LALES), an unrestricted departmental grant from Research to Prevent Blindness (LALES), DK-073541 (Starr County Health Studies), DK-085501 (Starr County Health Studies), DK-020595 (Starr County Health Studies), HL-102830 (Starr County Health Studies), and Consejo Nacional de Ciencia y Tecnología grants 138826, 128877, and CONACyT-SALUD 2009-01- 115250 (SIGMA Type 2 Diabetes Consortium, UNAM/INCMNSZ Diabetes Study).

Mexico City Studies

The Mexico City 1 and 2 studies of Mexican adults and the Mexico Case-control study of childhood obesity were supported in Mexico by the Fondo Sectorial de Investigación en Salud y Seguridad Social (SSA/IMSS/ISSSTECONACYT, project 150352), Temas Prioritarios de Salud Instituto Mexicano del Seguro Social (2014-FIS/IMSS/PROT/PRIO/14/34), and the Fundación IMSS. We thank Miguel Alexander Vazquez Moreno, Daniel Locia and Araceli Méndez Padrón for technical support in Mexico. In Canada, this research was enabled in part by two CIHR Operating grants to EJP, a CIHR New Investigator Award to EJP and by support provided by Compute Ontario (www.computeontario.ca), and Compute Canada (www.compute.canada.ca).

1982 Pelotas Study

The 1982 Pelotas Birth Cohort Study is conducted by the Postgraduate Program in Epidemiology at Universidade Federal de Pelotas with the collaboration of the Brazilian Public

Health Association (ABRASCO). From 2004 to 2013, the Wellcome Trust supported the study. The International Development Research Center, World Health Organization, Overseas Development Administration, European Union, National Support Program for Centers of Excellence (PRONEX), the Brazilian National Research Council (CNPq), and the Brazilian Ministry of Health supported previous phases of the study. Genotyping of 1982 Pelotas Birth Cohort Study participants was supported by the Department of Science and Technology (DECIT, Ministry of Health) and National Fund for Scientific and Technological Development (FNDCT, Ministry of Science and Technology), Funding of Studies and Projects (FINEP, Ministry of Science and Technology, Brazil), Coordination of Improvement of Higher Education Personnel (CAPES, Ministry of Education, Brazil).

San Antonio Mexican American Family Study

The San Antonio Mexican American Family Study was supported by HL045522, DK053889, DK047482, and MH059490.

Santiago Longitudinal Study

The Santiago Longitudinal Study was supported by the Eunice Kennedy Shriver National Institute of Child Health & Human Development (R01 HD033487-15), National Institute on Drug Abuse (R01 DA021181-05), and National Heart Lung, and Blood Institute (T32 HL079891-11). The Viva La Familia Study was supported by R01DK59264 and R01DK080457.

Mount Sinai Bio*Me* Biobank Program

The investigators of Bio*Me* 1 and 2 were supported by the following grants (MG-M: 0052155282325, MP: 0057 320 7034343, CS: 0055 51 999523134, RAJS: 0052153501900).
